# Cost-Efficient RSSI-Based Indoor Proximity Positioning, for Large/Complex Museum Exhibition Spaces

**DOI:** 10.3390/s25092713

**Published:** 2025-04-25

**Authors:** Panos I. Philippopoulos, Kostas N. Koutrakis, Efstathios D. Tsafaras, Evangelia G. Papadopoulou, Dimitrios Sigalas, Nikolaos D. Tselikas, Stefanos Ougiaroglou, Costas Vassilakis

**Affiliations:** 1 Digital Systems Department, University of the Peloponnese, GR-23100 Sparta, Greece; koutrakis@uop.gr (K.N.K.); stathistsafaras@gmail.com (E.D.T.); euaggelia.papadopoulou77@gmail.com (E.G.P.); sigalasd7@gmail.com (D.S.); 2 Informatics and Telecommunications Department, University of the Peloponnese, GR-22100 Tripoli, Greece; ntsel@uop.gr (N.D.T.); costas@uop.gr (C.V.); 3 Department of Information and Electronic Engineering, International Hellenic University, GR-57400 Thessaloniki, Greece; stoug@ihu.gr

**Keywords:** large/complex museums, indoor proximity positioning, Bluetooth Low Energy, RSSI temporal/spatial methods, machine learning classification, museum visitor modeling

## Abstract

RSSI-based proximity positioning is a well-established technique for indoor localization, featuring simplicity and cost-effectiveness, requiring low-price and off-the-shelf hardware. However, it suffers from low accuracy (in NLOS traffic), noise, and multipath fading issues. In large complex spaces, such as museums, where heavy visitor traffic is expected to seriously impact the ability to maintain LOS, RSSI coupled with Bluetooth Low Energy (BLE) seems ideal in terms of market availability, cost-/energy-efficiency and scalability that affect competing technologies, provided it achieves adequate accuracy. Our work reports and discusses findings of a BLE/RSSI-based pilot, implemented at the Museum of Modern Greek Culture in Athens, involving eight buildings with 47 halls with diverse areas, shapes, and showcase layouts. Wearable visitor BLE beacons provided cell-level location determined by a prototype tool (VTT), integrating in its architecture different functionalities: raw RSSI data smoothing with Kalman filters, hybrid positioning provision, temporal methods for visitor cell prediction, spatial filtering, and prediction based on popular machine learning classifiers. Visitor movement modeling, based on critical parameters influencing signal measurements, provided scenarios mapped to popular behavioral models. One such model, “ant”, corresponding to relatively slow nomadic cell roaming, was selected for basic experimentation. Pilot implementation decisions and methods adopted at all layers of the VTT architecture followed the overall concept of simplicity, availability, and cost-efficiency, providing a maximum infrastructure cost of 8 Euro per m^2^ covered. A total 15 methods/algorithms were evaluated against prediction accuracy across 20 RSSI datasets, incorporating diverse hall cell allocations and visitor movement patterns. RSSI data, temporal and spatial management with simple low-processing methods adopted, achieved a maximum prediction accuracy average of 81.53% across all datasets, while ML algorithms (Random Forest) achieved a maximum prediction accuracy average of 87.24%.

## 1. Introduction and Relevant Literature

With the advent and rapid development of the internet of things (IoT), Location-Based Services (LBS) are widely applied in several sectors, such as smart cities/buildings, with terrestrial indoor positioning technologies and systems emerging as a solution to the limited effectiveness of the popular Global Navigation Satellite Systems (GNSS), such as the Global Positioning System (GPS) [1,2]. Unlike satellite, indoor positioning systems have not yet been standardized, mainly due to the wide range of application areas, and different requirements stemming from diverse environments. Consequently, indoor positioning solutions tend to be highly deterministic, i.e., designed and customized for specific areas/environments. An important sector affected, is the culture industry, demonstrating increasing attendance and transformation of the way museum managers provide a cultural experience to visitors, evaluate visitor behavior, and correspondingly manage the cultural content [3,4]. In this context, museums are well-suited grounds for indoor-positioning experimentation.

Received Signal Strength Indicator (RSSI)-based proximity tracking is a well-known signaling technique for providing indoor location, featuring simplicity and cost-effectiveness, as it requires low-price, off-the-shelf hardware; however, it suffers from low accuracy in non-line-of-sight (NLOS) situations, experiencing noise and multipath fading issues [5,6]. In large, complex exhibition spaces and museums, where high visitor traffic is expected to be seriously impacting the ability to maintain line-of-sight (LOS) between the users’ devices and fixed tracking equipment, RSSI seems more appropriate considering parameters such as market availability, cost-efficiency, computational demand, scalability, and environmental sensitivity, negatively affecting other NLOS signaling techniques such as Channel State Information (CSI with relatively low market availability, requires significant computational resources, advanced algorithms, and calibration overheads, while being sensitive to environment dynamics and diversity) [7,8,9] and localization methods such as Fingerprinting (FP requires time-consuming and costly site surveys at initial setup, significant storage/computational resources for large-scale environments, while being sensitive to environment and device changes) [10,11,12]. All other basic position-dependent signaling techniques, require LOS and complex expensive equipment to achieve high accuracy. These include Time of Flight/Arrival (ToF/ToA), Time Difference of Arrival (TDoA), and Return Time of Flight (RToF) techniques, requiring also moderate to strict synchronization between devices [5,13,14], and Angle of Arrival/Phase of Arrival (AoA/PoA) techniques, requiring multiple antenna arrays and precise synchronization between antennae, thus increasing implementation complexity and cost [15,16].

Bluetooth Low Energy (BLE) technology, well-coupled to the RSSI technique, features low costs and power efficiency (inexpensive devices consuming minimal power), ease of deployment, and wide accessibility (BLE beacons are easily integrated in existing systems and ubiquitous in modern smartphones and IoT devices, thus facilitating seamless user adoption). BLE technology has the potential to apply sophisticated methods to enhance positioning accuracy, thereby reducing errors to the meter/submeter level in challenging indoor scenarios. Inherent limitations of BLE/RSSI-based indoor positioning include low accuracy (in ΝLOS situations), electromagnetic interference (EMI) due to the environment or other devices present, shadowing, and multi-path fading effects, which cause unpredictable signal distortion, and result in low robustness [5,6,17,18].

As environmental noise and static or moving obstacles are potentially present in all indoor environments, different algorithms have been applied to reduce their impact. Median/average filters (MFs/AFs), Kalman filters (KFs), and particle filters (PFs) are popular methods which have been proposed in the relevant literature [1,2,6,13,19,20,21]. While MFs/AFs are easy to implement, computationally efficient, and good at eliminating outliers, they cannot dynamically adapt to rapid changes, thus reducing robustness in dynamic environments. PFs use a probabilistic approach, representing the state of a system with a set of particles, and assigning weights based on how well particles match field data. While PFs work well in highly dynamic and nonlinear environments, such as those with intense shadowing and multipath fading, they require significant resources, especially with large numbers of particles, and seriously downgrade performance if particles fail to represent the state accurately (e.g., in dense NLOS situations). On the other hand, KFs use a predictive model to estimate the position by combining noisy RSSI measurements with system dynamics, continuously updating based on new measurements. Unless severe NLOS conditions occur, and considering accurate tuning (e.g., noise covariance), KFs handle noise more efficiently than simpler smoothing methods, and with less complexity compared to PFs, thus making them well-suited for moderately dynamic environments. It should nevertheless be noted that these issues are dynamic in nature and difficult to efficiently model, thus making field studies in real conditions better suited and clearly preferable to laboratory simulation.

Machine Learning (ML) has been extensively employed to address the inherent complexities of BLE/RSSI-based indoor localization and the limitations of traditional filtering techniques. ML algorithms such as k-Nearest Neighbor (K-nn), Decision Trees (DTs) Random Forest (RF), Support Vector Machines (SVMs), Bayes Net (Bayes-N) and Artificial Neural Networks (ANNs) enhance position prediction accuracy by learning complex patterns in RSSI data, replacing path loss modeling of signal attenuation, even in environments with non-linear noise, optimizing fingerprinting, and handling environmental variability [2,22,23,24]. The accuracy of ML models depends heavily on the quality and diversity of training data, limiting generalization across different environments. This essentially means that data collection and model training must be repeated for each new room layout and different museum architecture. Furthermore, ML algorithms often require large, labeled datasets for training, which can be time-consuming and costly to collect/process, while their real-time implementation in IoT, i.e., resource-constrained environments, can be challenging, due to high computational demands. This is especially true for deep learning solutions involving Convolutional Neural Networks (CNNs) and Long Short-Term Memory (LSTM) networks [2,22]. Despite their efficiency in addressing non-linearities, multipath interference, and signal fluctuations, CNN-/LSTM-based methods usually come with high computational costs and tuning requirements, typically requiring GPUs for training. In this context, their added value should be justified by the positioning accuracy improvement achieved versus other high precision, but expensive alternatives (such as visitor gaze detection, visual processing/recognition systems), and low precision, yet cost-efficient solutions (such as BLE). This issue becomes critical for large museum administrators, struggling to optimize museum performance and visitor experience, in terms of space coverage, system complexity, implementation, and maintenance costs [3]. It should also be noted that the quest for maximum accuracy may become less relevant when visitor whereabouts can be assumed/calculated from context data.

Despite the fact that the indoor tracking of humans is a dynamic and rapidly developing technical field with a plethora of both scientific and commercial applications, only a limited number of studies can be found in the recent literature reporting field pilots from large/complex museums/exhibition spaces, employing cost-efficient solutions that are comprehensively documented and leveraging both museum performance and visitor experience.

In [25], Romeo Giuliano et al. (2020) analyzed an indoor localization system based on BLE in a museum environment, using RSSI to estimate the position of the visitors’ devices, transmitting signals to BLE receivers deployed strategically in the museum area. Results showed an accuracy below 1 m, using a feed-forward neural network and a non-linear least square algorithm. However, there was no quantification of position prediction performance, while the actual pilot site is limited to a single room and not linked to a specific museum. In [26], Petros Spachos and Konstantinos Plataniotis (2020) analyzed the placement of BLE beacons in an IoT-based interactive smart museum. Kalman filters implemented in the visitor smartphone were used to improve RSSI accuracy, without the need of the cloud. Experimental results indicated that the BLE beacons’ placement and density determine detection accuracy (as the distance between neighboring beacons increases, detection accuracy is more challenging and, as the number of the neighboring beacons increases, detection accuracy decreases). However, all experiments were conducted in a laboratory environment. In [27], Richard Jérémy et al. (2021) analyzed an indoor system based on the proposition of a minimal zone searching (MZS) algorithm applied to visitor paths within a French museum, promoting the use of less intrusive, cheap, wearable BLE beacons, as opposed to visitor smartphones. Results showed that, with the MZS algorithm, the position of sensors seems to have less impact, i.e., spaces with few sensors are better tracked, making MZS specifically useful in tight spaces. However, the algorithm alone returns a zone and not an adequate position accuracy, i.e., requiring to be coupled with an additional method, while sensor placement is found to have critical impact on accuracy. Only a limited number of sensors and rooms were used in experiments, and two datasets were processed, an open-access one, and one recorded on-site during a visit by high school students. In [28], Alexandros Kontarinis et al. (2021) presented a new conceptual model of trajectories to account for semantic and indoor space information, supporting the design and implementation of context-aware mobility data mining and statistical analytics methods. A case study was implemented on Louvre Museum’s visiting data, showcasing how state-of-the-art mining algorithms can be applied on trajectory data represented according to the proposed model, and outline their advantages and limitations. An extended BLE beacon infrastructure (1800 beacons in all five floors of the museum) was used, offering RSSI-based tracking, improved with extended KF and PF noise reduction techniques. Visitor smartphones were used, leading to partial recordings, and thus sparse movement datasets, as visitors would launch/close the application mid-visit.

With regards to visitor behavior modeling, in [29], Yuji Yoshimura et al. analyzed visitor sequential movements, the spatial layout, and the relationship between them in large-scale art museums—a case study for the Louvre Museum—using anonymized data collected through non-invasive Bluetooth sensors. The analysis revealed that time (short visit vs. long visit) does not significantly differentiate visitor behavior, while the study of unknown behaviors is key to improving the visiting experience. One of the earliest taxonomies in the relevant literature is [30], where Veron and Levasseur identified four different visiting styles (based on the way they interact with artworks) using metaphors from animal motion behaviors (ant, fish, grasshopper, and butterfly), which seems to have survived via adoption and/or extension in numerous subsequent studies, e.g., by focusing on social interaction or other aspects [31,32,33,34].

In our current work, we report and discuss the findings of a BLE/RSSI-based indoor proximity positioning pilot, implemented at the premises of the Museum of Modern Greek Culture (MNEP) [35], in the framework of the MELTOPENLAB R&D project [36], elaborating on the functionality and implementation of a visitor tracking tool (VTT), following the preliminary results reported in [3]. The VTT functional architecture is proposed as a comprehensive indoor proximity positioning framework, integrating different positioning accuracy enhancement methods. Its pilot implementation follows the overall MELTOPENLAB concept, the adoption of available, affordable, and scalable solutions, and low complexity methods in an IoT context, achieving an optimal trade-off between technical performance and cost efficiency, targeting large/complex museums/cultural organizations, with high anticipated visitor foot traffic.

The article’s organization is as follows:

Section 1 provides the introduction, scope, the relevant literature and background studies supporting the assumptions and implementation decisions in the current work considering indoor positioning in the culture industry, RSSI and competing proximity tracking techniques, BLE beacons, noise filtering techniques, and machine learning algorithms for position prediction. Section 2 presents the overall system and the visitor tracking tool (VTT) functional architecture, followed by the pilot setup description in a bottom-up manner, from site areas (MNEP), sensor infrastructure, and coverage implementation to critical parameters and assumptions regarding the visitor modeling and movement patterns used for RSSI measurements. Section 3 continues with the VTT functionalities implementation, including data collection and denoising, RSSI temporal and spatial management, and ML classification, along with the description of datasets built from on-site RSSI measurements, and the accuracies achieved by different positioning prediction methods over these datasets. Section 4, discusses techno-economic issues, system pilot parameters, and their impact on measurements, and evaluates the performance of temporal, spatial, and ML methods, providing an overall ranking of methods. Section 5 assembles the conclusions and reports future research plans.

## 2. System Architecture and Pilot Setup

The MELTOPENLAB system architecture, with an emphasis on the visitor tracking component, and the critical MNEP pilot setup considerations and assumptions are presented and discussed in the following, providing analysis on sensor allocation and visitor movement modeling.

### 2.1. Functional Architecture

As introduced in [3], the MELTOPENLAB addresses holistically and integrates four important aspects relevant to enhancing both visitor experience and museum performance:

(a) integrated digital documentation of exhibits in terms of four complementary ontologies, including their methods for conservation and restoration,
(b) a tool prototype for tracking visitor proximity to exhibits in near real-time (nRT), in both an eponymous and anonymous manner,
(c) a mobile application for creating and providing personalized presentation, entertainment, and learning experience to visitors, and
(d) a platform for museum administrators to manage visitor tracking and rating statistics for the evaluation of exhibits.

The overall concept, focuses on cost efficiency and scalability, matching the requirements of a multi-space and high-traffic modern museum.

**Figure 1:**
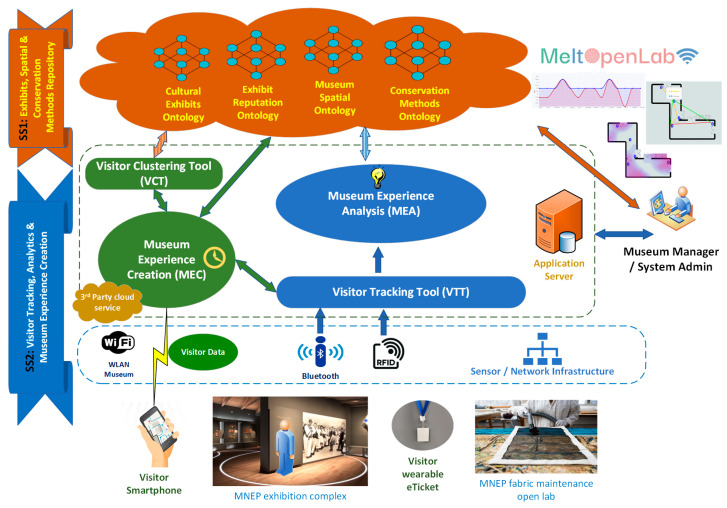
MELTOPENLAB generic functional architecture.

Figure 1 depicts the layers and individual operating components of the MELTOPENLAB generic functional architecture, corresponding to the aforementioned aspects.

The overall functionality is divided into two subsystems, one (SS1) related to the organization, storage, and management of exhibits and visitor data/metadata (four ontologies supported); the other (SS2) for managing visitor tracking (in the VTT), content presentation via a mobile application (MEC), customized (VCT) on the visitors’ static (demographic) and dynamic (behavior) characteristics, and a museum administrators front-end (MEA) for managing/visualization of visitor data analytics.

The MELTOPENLAB system architecture and components depicted in Figure 1, have been analytically presented and discussed in [3], so the focus in this sequel, will be on the VTT, a middleware that tracks museum visitors based on Bluetooth Low Energy (BLE) RSSI time series data.

**Figure 2:**
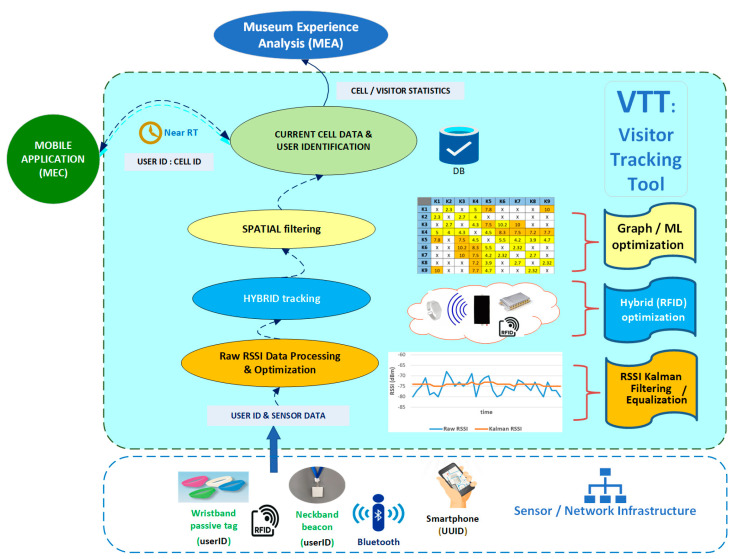
Visitor Tracking Tool (VTT) generic functional architecture.

Figure 2 depicts the generic functional architecture of the VTT, providing a comprehensive framework for optimizing localization efficiency and accuracy, consisting of functional layers, described in a bottom-up fashion, in the following. The lower layer includes arrays of sensors appropriately installed in diverse museum exhibition spaces, defining cells of coverage, corresponding to thematic sets of exhibit showcases, according to museum requirements (e.g., exhibition concepts, aesthetic constraints) and other technical limitations (e.g., availability of power outlets near showcases).

The detection of a visitor’s presence in a cell is determined by the visitor’s eTicket beacon signal strength, as received by surrounding sensors, with appropriate lower thresholds (ranging between −70 dBm and −90 dBm for different MNEP halls), thus implementing a proximity-tracking mechanism, supporting near real-time cell-level location detection, with cell updates ranging between 2 and 4 s, relevant to visitor movement modeling.

Suffering from intense fluctuations due to unpredictable interferences and severe signal distortion/degradation in closed spaces with static obstacles and moving humans, and creating an NLOS environment with significant shadowing and multipath fading effects, RSSI raw data are denoised/equalized by applying appropriate Kalman Filtering. Optimized/smoothed RSSI data, with values that are now considered more reliable, are used to determine the visitor’s cell by processing appropriately timestamped sets of RSSI values (records in the VTT database).

Provision for additional technologies that may complement/enhance original single-technology RSSI-based positioning accuracy is supported in the next level. Different hybrid schemes may be employed in this context. Radio Frequency Identification (RFID), being a MELTOPENLAB candidate technology along with BLE, was integrated into the system design as a hybrid technology to increase cell detection accuracy, using low-cost short-range wearable RFID passive tags (eTickets) for visitors.

Museum topology considerations related to visitor potential movement are integrated as an additional level of position filtering, based on spatial knowledge. Different methods may be applied at this level, including graphs of allowed transitions, utilizing static (e.g., cell allocation/relationships), as well as dynamic (e.g., visitor behavior modeling) information, effectively spatial hints customized to specific museum spaces in a deterministic manner.

Finally, at the top level, the use of ML algorithms, integrating in their training pilot RSSI measurements and potential hints provided by the above graphs, that can be static (e.g., spatially nearest cells) or dynamic (e.g., next cell when following a certain route/visitor model), may provide further improvements to cell prediction accuracy. As already noted in the Section 1 literature presentation, ML is instrumental in learning complex RSSI data patterns, replacing path loss modeling of signal attenuation, optimizing fingerprinting, and handling environmental variability.

As depicted in Figure 2, the VTT informs the rest of the SS2 components (MEC, MEA) regarding the visitor’s current cell in both the eponymous and anonymous mode of operation (analysed in [3]). As already introduced in Section 1, the VTT’s functional architecture forms a comprehensive indoor positioning framework, integrating different accuracy enhancement methods.

The following subsections elaborate on the methods and assumptions adopted in the MELTOPENLAB VTT pilot implementation.

### 2.2. MNEP Exhibition Spaces

The Museum of Modern Greek Culture, is the largest contemporary public museum in Greece, dealing since its original establishment, in 1918, with the tangible and intangible aspects of modern Greek cultural heritage, rescuing, studying, and highlighting everyday life and ritual symbols and objects (approx. 3000 cultural exhibits), as well as information on morals and customs, traditional arts, and techniques. It is situated in a multi-building (11 exhibition and 9 support spaces) complex in Monastiraki, one of the most touristic spots near the center of Athens.

**Figure 3:**
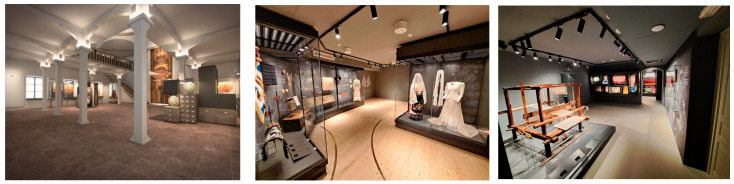
Museum of Modern Greek Culture indicative indoor views.

Figure 3 depicts indicative indoor views of the MNEP building complex.

The exhibition spaces of the MNEP complex present a wide range of sizes, ranging from small square/rectangular halls with an area of 9–10 m^2^ to large, often narrow halls with an area of 55–70 m^2^, with varying openings (windows, doors), surfaces (walls, showcases), and densities of intermediate obstacles. A unique, in terms of hall characteristics, case, is the Tsisdaraki Mosque (Tzami), a single bell-shaped hall with an area of 105 m^2^.

The criteria for building/hall selection included adequate representation of different hall sizes, shapes, and surfaces, as well as practical issues, such as the potential to power devices (existing outlets and wiring), the MNEP aesthetics (allowed interventions), and stage of preparation (installation of sensors for halls that were under construction).

Eight (8) buildings with 47 halls were selected for the MELTOPENLAB pilot, summing up to a total area of 1372.29 m^2^. The distribution of the exhibition room areas (in m^2^) in the eight buildings covered is presented in following Table 1, with appropriate color-code visualizing the hall areas’ diversity, ranging from red (small) to green (large) spaces.

Characteristics of the halls under coverage, associated with factors affecting radio coverage and sensor operation, are listed in the sequel.

- *Surfaces and openings*: Walls, doors, furniture, and showcases absorb radiation, reducing power during propagation. Reflective surfaces can cause distortions with increased multipath fading, while openings seriously impact such phenomena. The under-coverage areas were characterized by a multitude of windows/doors and, for the most part, reflective surfaces (metal/glass showcases, smooth walls).
- *Line of Sight and Shadowing*: Lack of LOS (NLOS), as well as objects between transmitter and receiver (shadowing), cause degradations in signal strength. The under-coverage areas were generally characterized by an absence of intermediate obstacles, except for some large rooms (about 20% of total) with centrally placed showcases. In addition to static, dynamic shadowing, associated with the volume and movement of visitors, was assumed to be high for the MNEP.
- *E/M Interference*: Electronic devices of all kinds may interfere with and distort signals. The areas covered were generally characterized as typical static, bearing fixed lighting and networking (security, WLAN) equipment, and high dynamics associated with the density and movement of the visitors’ devices (smartphones).

### 2.3. Electronic Tickets and Sensors

As explained in [3], smartphones cannot effectively and efficiently cover the requirements of visitor tracking for a number of reasons:

(a) visitors may not activate Bluetooth, or even use a mobile device during their visit, regardless of the incentives provided by the museum;
(b) Android and iOS MAC ID randomization make tracking almost impossible, as the Bluetooth MAC address changes periodically [37];
(c) mobile devices’ power-saving schemes significantly degrade transmitted signal stability, a prerequisite for accurate RSSI-based detection/tracking.

Visitor e-ticketing solutions that are popular among museum managers for extending the potential of security and statistics collection were adopted in the MELTOPENLAB system [3] to address the above user-device (smartphone) shortcomings. BLE beacons and RFID ARPTs (active reader passive tags), in the form of wearable, low-cost neckbands and wristbands, respectively, were selected for experimentation, ensuring a stable, detectable signal.

Specifications relevant to coverage and power efficiency for the BLE wearable e-ticket, which was the basic user device in the MELTOPENLAB pilots, and the corresponding sensor (BLE Gateway) are listed in Table 2.

### 2.4. Signal Thresholds and Cell Coverage

Different MELTOPENLAB pilot phases were conducted with appropriate parametrization of the sensors’ signal thresholds, thus defining the BLE gateways’ reception sensitivity. Coupled with BLE beacons (visitor eTickets) with the transmission power level set at −12 dBm, the BLE gateways’ placement defined virtual cells of coverage for proximity detection. Cell size was adopted to spatial constraints, relevant to room size/shape and showcase distribution, with the criteria discussed in Section 2.2. In most cases, BLE cells would correspond ideally to a semi-circular area of about 12 m^2^, defined by a 3 m radius around a wall-mounted BLE gateway. Considering the specifications of BLE equipment and the overall profile of E/M noise in the MNEP halls, a lower threshold of −80 dBm was deemed adequate for reliable RSSI measurements in all cases, except for the Tz building (Tsisdaraki Mosque), where a more complex threshold scheme was used, due to different type of sensor installations. Table 3 provides the thresholds used per building and pilot phase (P1, P2, P3, P4, R1/R2).

As already mentioned in Section 2.2, continuous changes in the layout of the exhibition spaces, as well as restrictions in allowed interventions to showcases and wall-mounted power supply points were the main factors constraining optimal installation of sensors.

On the other hand, for most exhibit collections, there was a semantic connection between showcases/exhibits, resulting in the formation of clusters/groups of showcases/exhibits and their assignment to cells, being both a requirement from the MNEP and feasible in most cases (with regards to composing and presenting relevant content).

**Figure 4:**
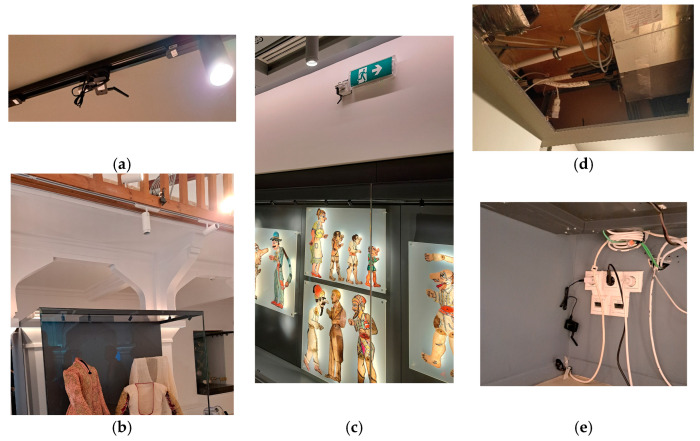
BLE gateway installation types in the MNEP: (**a**) ceiling lightning bars (ERCO), (**b**) mounted on wooden bars, (**c**) wall-mounted on emergency light, (**d**) within false ceiling, and (**e**) under the showcase.

Figure 4 depicts different types of BLE gateway installations.

**Figure 5:**
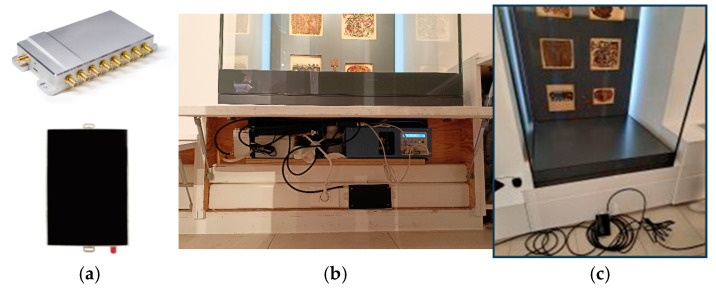
RFID equipment installation in the Tz (Tsisdaraki Mosque): (**a**) reader and antenna, (**b**) antenna and cables vs. showcase, (**c**) installation in wooden locker.

Regarding the RFID equipment, as the readers (Rodinbell, Spider 8000), antennae (Kreet D865C03P, 3dbi, Circular), and cables (Rodinbell 10M5DFBSM) for their connection were quite bulky and difficult to mount near most showcases, it was installed only in building Tz (Tsisdaraki Mosque), inside the wooden lockers under seven of the nine showcases. The transmission power of the RFID-reader antennae (directional with an angle of 60°) was set to −31 dBm, the maximum supported by the specific equipment, which corresponds to a coverage area of approximately 0.5 m^2^ (circular sector of radius 1 m and angle 60°). Figure 5 depicts the RFID equipment installation in the Tz.

RFID and BLE Gateways communicated positioning data via WiFi access points implemented as a wireless transport, autonomous from other museum WLANs/networks.

According to the mapping between the MNEP exhibits (showcases) and cells (BLE gateways) carried out in MELTOPENLAB [3,36], approximately 130 physical and digital exhibits were mapped to 64 cells, corresponding to a mean density of about two showcases per cell. Regarding the average distribution of sensors in the under-coverage areas, the only hall with relatively dense sensor placement (one BLE gateway/11.69 m^2^) was the Tsisdaraki Mosque; while, in all other cases, the average spatial density of sensors was one BLE gateway/23 m^2^ (ranging from 16.27 to 34.62 m^2^ per building), allocated in correspondence with the thematic grouping of exhibits/showcases and blind areas not covered with BLE cells.

**Figure 6:**
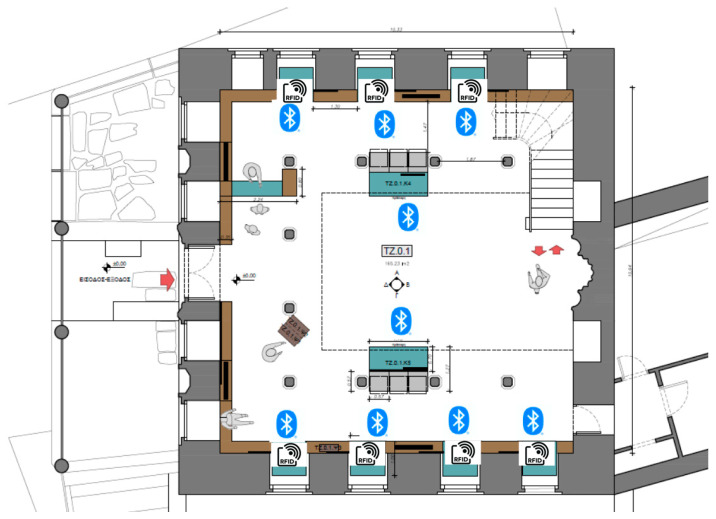
BLE and RFID sensors allocation in the Tsisdaraki Mosque.

Figure 6 below depicts the floor plan of the Tsisdaraki Mosque with the distribution of BLE and RFID sensors.

### 2.5. Visitor Movement Modeling

Modeling of the visitor movement in the MNEP spaces was based on a series of factors, some already identified (Section 2.2) as critical parameters influencing signal measurements (i.e., RSSIs) during propagation into museum spaces:

➢ *Visitor density*, the *proximity concept*, defined by the implementation of sensor cells in the halls and the presence of the visitor e-ticket inside/outside these coverage areas determining detection, provides unclear cell boundaries with inevitable overlaps. Contact (*LOS/NLOS*) between the detection device (e-ticket) and the sensor defining a cell is influenced by obstacles, both static (showcases) and moving (visitors). Given the size of the cells (BLE cells: 12 m^2^), the average size and density of showcases per cell (approximately 2 showcases per cell) and the expected high traffic of the MNEP, we assumed an average number of four visitors per cell during peak hours.
➢ *Visitor speed*, which practically affects the rate of cell change/roaming, depends on many factors, the main ones being the density and layout of the showcases, and the demographics/types of visitors. While general pedestrian walking speeds are around 1.4 m/s, museum visitors typically move at a slower pace to engage with exhibits [38]. We assumed an average of 1.2 m/s, which corresponds to 4.32 km/h.
➢ *Cell roaming rate*, is the average rate of movement between cells (including cell revisits) as a function of the average speed of movement and the average time spent in a cell. The average time spent in front of an exhibit can vary widely depending on the type of exhibit, the level of interest and, more generally, the behavior (engagement) of the visitors, the density of exhibits, and of the visitors. While commonly accepted values for average time spent in front of an exhibit in art museums range from 21 s [39] to more than 41 s [40], given the density of exhibits and the high expected traffic in the MNEP, we assumed a lower threshold of 10 s to model the minimum time spent looking at an exhibit in a cell, and accordingly adjusted the system response for optimal operation in peak hour conditions. If the average distance between (the centers of) two adjacent (and marginally overlapping) cells is about 5 m to 6 m, a visitor moving at an average speed of 1.2 m/s needs approximately 4.6 s to change cells. With an average of two showcases per cell, a visitor would spend at least 20 s in the cell looking at exhibits, and the average cell roaming rate would be 24.6 s. In this context, a visitor cell roaming rate is considered slow, or fast, as compared to this number.
➢ *Navigation mode* refers to structured (pre-determined static route/specific mode of movement) or unstructured (free movement without restrictions on movement inside/outside the cells) roaming between exhibits. We defined four navigation modes, with different degrees of freedom of movement:
  ○ *Nomadic movement* with structured, or free movement from cell to cell and adequate time to examine the exhibits (e.g., as part of a guided tour, or a predetermined route), which implies a relatively slow cell roaming rate, and a high rate of visitor and sensor contact (LOS).
  ○ *Continuous movement* with structured or free movement between cells without systematic stay in cell to examine exhibits, which implies a relatively fast average cell roaming rate and low LOS (i.e., mainly NLOS) contact between the visitor and the sensors (mainly outside and at the boundaries of the cells).

Table 4 summarizes the critical settings of the above factors, from the combinations of which there emerge three different visitor movement models. 

These correspond to three basic models (profiles) of visitor behavior, as discussed in Section 1, captured by the relevant older and more recent literature [30,31,34]. The “ant” visitor follows specific paths (usually linear ones and at a short distance from the displays) and meticulously observes the exhibits, in contrast to the “butterfly” visitor, who follows an irregular path, frequently changing direction of movement and stopping to observe the exhibits without specific priorities, but with interest in almost everything. By contrast, the “fish” visitor often moves to the center of the space without focusing on the exhibits, choosing mainly those that are not crowded and focusing on the broader picture/location of the exhibits within a hall without seeking specific information (from applications, tour guide, etc.).

The above models (effective movement scenarios) do not exhaustively cover all expected visitor behaviors, but they do incorporate a key variation from less to more degrees of visitor moving freedom, and verify a basic requirement for the system to timely and effectively respond to changes in the detection area (cell), in terms of a few seconds, to adequately cover movement between cells.

In the context of visitor movement scenarios/models, timing is critical for managing the sampling rate of the RSSI values, which in turn determines the reliability of the smoothing/equalization filters applied. If the sampling rate of the RSSI values is sufficiently faster than the rate of visitor cell change (min cell roaming rate 4.6 s), then we can, with relative confidence, attribute significant RSSI fluctuations to noise, rather than visitor cell roaming. In other words, when the visitor is static, or nomadically moving, sudden and isolated fluctuations of the RSSI values from the sensors covering the area can be safely attributed to noise, such as shadowing, multipath fading, and interference.

Given sufficient smoothing of fluctuations and consequently increased reliability of signal strength values, when managing RSSI measurements to determine visitor current cell, the maximum RSSI value (or any other indicative combination of RSSI values) can be searched for within a time window defined by the temporal discreteness of the RSSI measurements (max BLE gateway sampling rate ≈ 1 s) and the expected cell roaming rate of the visitors (min. 4.6 s, average 24.6 s, max. 45.6 s).

### 2.6. Movement Scenarios and Measurement Patterns

As already discussed in Section 2.5, we assumed an average number of four visitors per cell during peak hours. As a comprehensive approximation of real-life conditions, such a group of visitors would exhibit both static (e.g., still in front of a showcase) and dynamic (e.g., changing directions) behaviors, while staying in the cell, in nomadic movement scenarios, corresponding to the visitor models “ant” and “butterfly” in Table 4. We mapped such behaviors to movement patterns, defining the discrete visitor roles as follows:

☺ “Visitor”: moving (changing view/direction) within the cell in a nomadic manner and at low speeds (at the order of 10 s per view/direction)
☺ “Crowd”: moving (changing view/direction) within the cell continuously and at moderate to fast speeds, shadowing the “Visitor”.
☺ “Body”: standing almost motionless, at the boundaries and within the cell area, part of the time close to the “Visitor”.

**Figure 7:**
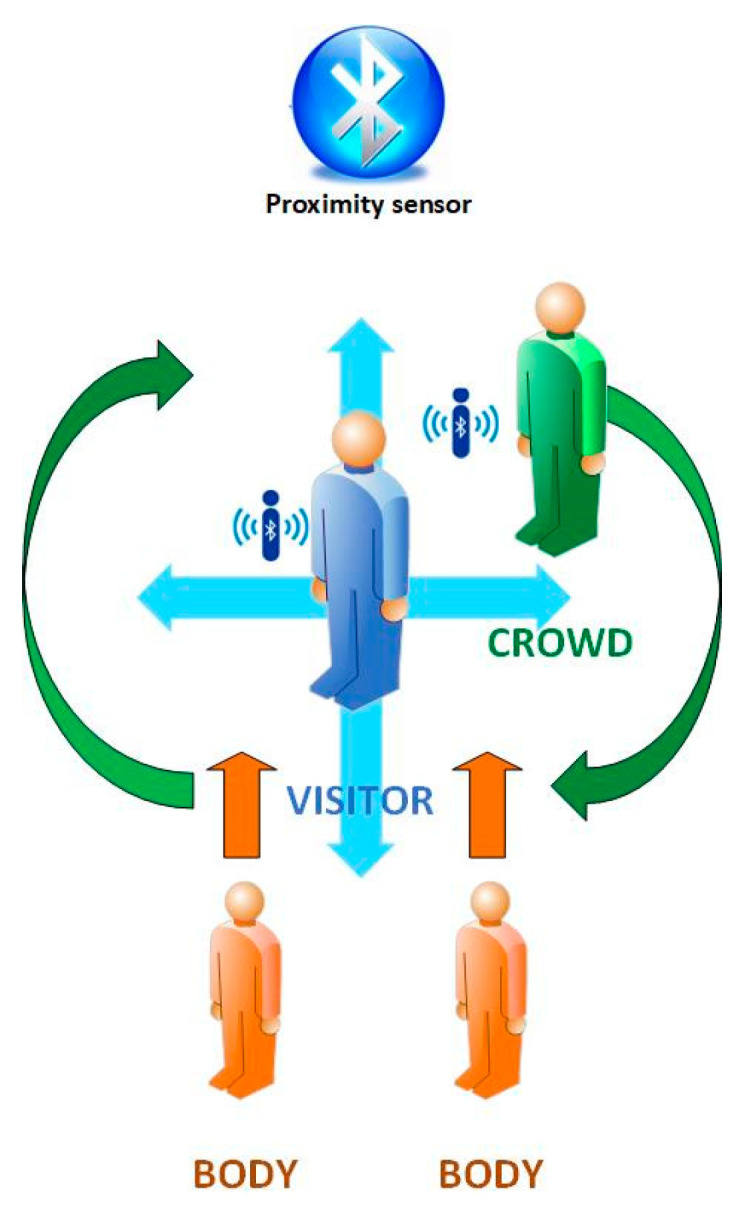
Measurement pattern with four users and three roles (“pattern-4”).

Based on the above roles, we defined specific movement scenarios for the implementation of the pilot measurements, such as the following “pattern-4”, illustrated in Figure 7:

A “Visitor” user stands in the middle of the cell, looking at a showcase and performs successive rotations clockwise, with 90° steps every 10 s. The user is thus positioned in four different directions with respect to the sensor and the coverage area, without interference from other users in the cell.

Once a full circle is completed, the “Visitor” repeats the rotation, with the addition of a “Crowd” user, which circles around in close proximity shadowing the “Visitor”, and completing a full orbit with each change of direction of the “Visitor”. Upon completion of the second rotation of the “Visitor”, two “Body” users, standing relatively still from the beginning within the boundaries of the cell, but further back without interfering, now approach and all roles remain close together and still, looking at the showcase.

The total time spent in the cell is in the order of 90–100 s, corresponding to a slow cell roaming rate (Table 4). Clearly, the main active user in “pattern-4” is the “Visitor”, and secondarily the “Crowd”, while the “Body” roles are subsidiary, they are used to create more realistic shadowing conditions by simulating the presence and movement of a small crowd around the main measurement point.

A simpler variation of the above measurement pattern, involving only the “Visitor” and “Crowd” roles (“pattern-2”), aims to simulate low traffic conditions in the cells.

A critical parameter that may be differentiated in the above patterns is the execution time of the pattern, which is reduced to half (5 s stops). In this way, the following two basic nomadic movement scenarios are formulated:

- Nomadic_Structured: corresponds to “ant” visitor and pattern-4 slow (with 10 s stops)
- Nomadic_Free: corresponds to a “butterfly” visitor and pattern-4 fast (with 5 s stops)

To capture the continuous movement of users without a systematic stay inside cells while roaming, two cases can be distinguished, corresponding to structured and non-structured cell roaming scenarios. The number of visitors participating in each scenario can have a lower bound, related to the number of eponymous visitor types (six types supported in the MELTOPENLAB concept [3]) and the average size of a cell:

- Continuous_Structured: groups consisting of at least six visitors with smartphones (MEC application) and e-tickets (BLE neckbands) follow a predefined tour of part of the museum spaces with no particular movement restrictions inside/outside the cells, other than following an exhibit visit plan (in cell areas) corresponding to a static (predefined by the MNEP) route.
- Continuous_Free: groups consisting of at least six visitors with smartphones (MEC application) and e-tickets (BLE neckbands) follow a free tour of part of the MNEP areas without any particular movement restrictions inside/outside the cells.

## 3. Pilot Implementation and Measurements

The pilot tests of the MELTOPENLAB were carried out in five phases, with a gradual scaling of both the coverage (number of buildings participating in the tests) and the verification of functionalities (in tools, applications, DBs, and interfaces) of the overall system. Table 5 summarizes the five phases of system testing with indicative reference to the total number of RSSI records in the VTT DB, buildings covered, and measurement patterns used.

A total of 34 datasets were produced, using the 2-role and 4-role measurement pattern (pattern-2/4), as described in Section 2.6, including (in different datasets) both “Visitor” and “Crowd” role records. This was justified, considering that the “Crowd” role, as it incorporates both continuous directional changes and static intervals in front of exhibits, constitutes a variant of the “Visitor” role, with an interval of dynamic and fast movements within the cells. On the other hand, “Body” roles were not recorded, as these roles are ancillary, being used to create more realistic shadowing conditions (NLOS) from the presence of visitors around the small measurement area in which “Visitor” and “Crowd” roles are moving. All these datasets correspond to *Nomadic_Structured* and *Nomadic_Free* movement scenarios, as described in Section 2.6. However, due to field implementation issues (mainly in the P4 phase), including equipment failures (such as BLE gateway malfunctions), last-minute MNEP changes in the showcases (affecting cell allocation), and problems with the MNEP connectivity to the internet (affecting RSSI timestamps), only 20 datasets were considered valid for further processing and production of meaningful results. The datasets retained incorporate a representative mix of diverse halls in seven of the eight buildings and are relevant to the *Nomadic_Structured/free* scenarios. The other two movement scenarios (*Continuous_Structured/Free*) were out of scope, as they pose challenges that increase complexity, such as the definition of current cell while roaming continuously over cell boundaries, and the meaningful provision of location-based content. Table 6 summarizes the characteristics of the datasets in terms of the MNEP areas, number, density, thresholds of BLE gateways, number and density of records (RECs), and measurement pattern This is discussed in further detail in Section 4, in relation also to the other tables in Section 3.

**Figure 8:**
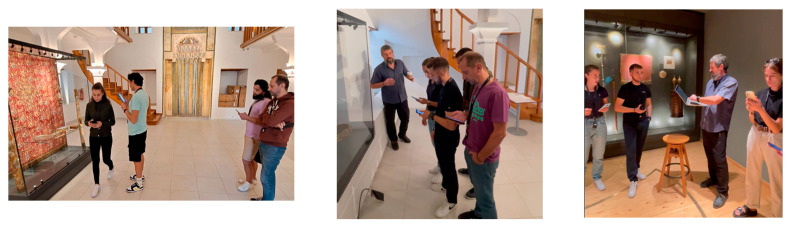
UoP team carrying out measurements in Tzami Tsisdaraki and building A.

Figure 8 depicts indicative instances of the UoP project team carrying out pattern-4 measurements in Tzami Tsisdaraki and Building A.

The following sections provide a detailed description of the pilot implementation, methods adopted, and results captured in the above 20 datasets, following a bottom-up approach with regards to VTT functionality, as introduced in Section 2.2.

### 3.1. Data Collection, Kalman Filtering, and Workflow

**Figure 9:**
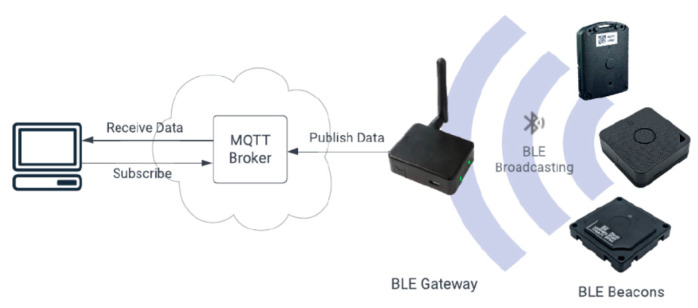
MQTT broker providing publish–subscribe messaging for the BLE data.

The asynchronous Message Queue Telemetry Transport (MQTT) lightweight protocol was adopted for feeding the BLE gateway data to the VTT, as it allows message distribution to be performed very quickly while minimizing traffic over the network, resulting in more efficient communication, reduced response times, and improved tracking [41]. An open-source MQTT broker (Eclipse Mosquitto broker version 1.6.9) was used, providing a cost-efficient, reliable, and secure message delivery for the BLE gateway data over the MELTOPENLAB WLAN installed in the MNEP, as illustrated in Figure 9.

Recording and management of sensor data for the VTT experimentation was implemented with a MySQL server, version 8.0.35. Raw data recorded in the VTT DB include unique timestamps, visitor beacon ID, cell (BLE gateway) ID, and RSSI value in dBm.

As introduced in Section 1 and Section 2.1, Kalman filters were adopted for providing an adequate level of signal equalization by reducing environmental noise (shadowing, multi-path fading, interference) to obtain more reliable signal power (i.e., RSSI) values. Due to the layout of the MNEP spaces (small to medium halls with many openings) and the highly reflective surfaces (Section 2.2), raw RSSI values obtained suffered from random and large fluctuations, making them practically unusable. Kalman filtering, being a recursive algorithm, considers previous RSSI measurements and produces an estimate of the true value by applying a prediction and correction procedure that minimizes noise. 

Its main advantages are that, due to its recursiveness, it can be applied to real-time measurements, and that it does not require any kind of initialization. The optimal VTT configuration involved experimental (in-field) balancing between the desired level of signal purity (RSSI error margin) and the speed of system response to visitor position changes (effectively cell roaming rate). To this end, we applied a one-dimensional linear Kalman filter, which is particularly effective for smoothing noisy measurements, assuming linear dynamics [42], and behaves well in the case of static objects and moving objects with low speed. 

As the sampling rate of the RSSI values obtained was set sufficiently high (one measurement per s) compared to the fastest adopted visitor cell roaming (4.6 s–see Section 2.5), it can be safely assumed that the visitor is not changing cells in this time window, as environmental noise in our measurements was usually larger than the difference in RSSI values created by visitor movement (nomadic mobility) obtained from neighboring BLEs. A separate 1D-Kalman filter was applied to each measurement stream per BLE Gateway. 

**Figure 10:**
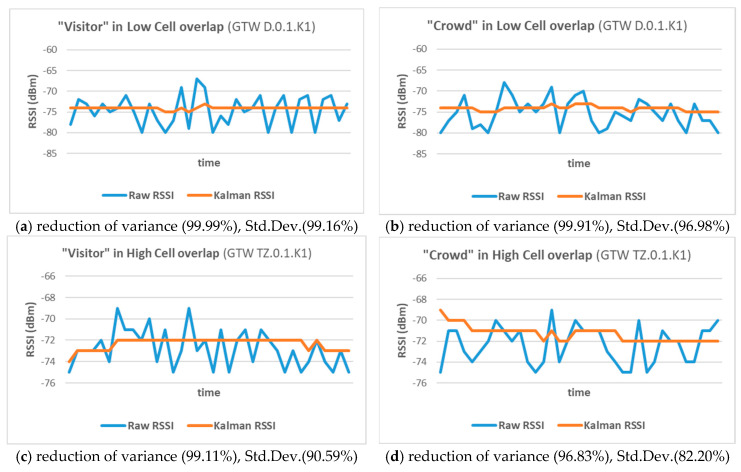
Smoothing RSSI values using Kalman filters (1D linear) in low/high cell overlap cases for “Visitor” and “Crowd” roles.

Figure 10, presents indicative diagrams, depicting linear 1D-Kalman smoothing over the random variations of the RSSI values corresponding to the movement of both visitor and crowd roles (see Section 2.6) in two cases representative of the MNEP halls:

- tubular space, with low cell overlap (gateway D.0.1.K1), and
- open space, with high cell overlap (gateway Tz.0.1.K1).

Raw RSSI values, before (blue) and after the application of KF (orange), demonstrated significant reduction of variance and standard deviation (Std.Dev.), which was more evident in the low overlap case.

**Figure 11:**
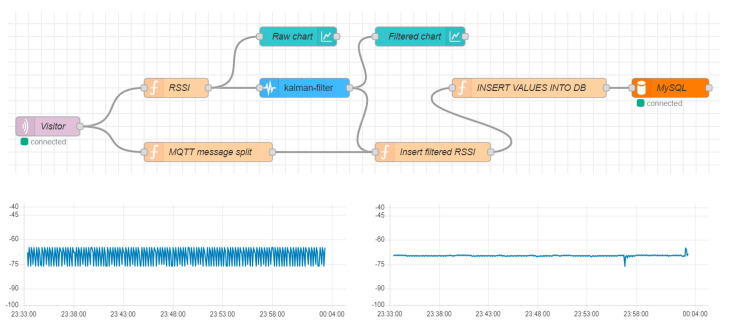
Node-RED environment indicative of the MNEP flow with raw/filtered data.

All VTT processes involved in receiving, processing, and storing data from the sensors, as well as deciding the current visitor’s cell, were based on data streams created using the Node-RED flow-based development tool (https://nodered.org/ accessed on 23 January 2025), accessed remotely to ensure seamless monitoring of the VTT operation. For each sensor message arriving at the server via the WLAN network, a series of processes were followed, resulting in its storage in the VTT database. An illustrative MNEP data flow in the Node-RED environment is depicted in Figure 11, including generation of raw chart and filtered chart (KF) diagrams.

### 3.2. RSSI Temporal Management

RSSI values that are considered more reliable, after KF smoothing, were used to determine the current visitor cell by searching for the maximum RSSI value (maxRSSI) between all RSSIs recorded for a given visitor beacon ID from all BLE gateways in each MNEP building. This simple method was adopted, considering the sufficiency of rough proximity (room-level presence detection in many of the MNEP halls) and the minimal computation required, and considering the overall low complexity/cost efficiency of the MELTOPENLAB concept.

In this context, three variations of calculating maxRSSI were tested, where the set of measurements (timestamped RSSI values from all building BLEs) considered included only the current second (one record), the last three records, and the last six records, named respectively maxRSS-1, maxRSS-3, and maxRSS-6. The rationale for these variations originated from the visitor movement modeling (Section 2.5), providing a range for the VTT operation between the fastest possible alternative (maxRSS-1 equal to the RSSI sampling rate) and more realistic alternatives (maxRSS-3 just under and maxRSS-6 just above the 4.6 s max. cell roaming rate). Thus, maxRSS-6 considers six records for each new prediction, five of which were reused in the previous one. Following an important observation, while making preparatory pilot measurements related to the “inertia” of system response, upon entering a new cell (positioning errors due to considering old cell records), a fourth method was tested (maxRSS-6c), a variation of the maxRSS-6, where the first five records upon entering a new cell are not considered. To provide further insights on the temporal behavior of the maxRSSI, a fifth method was applied (maxRSS-X), providing the peak maxRSSI prediction accuracy obtained from a curve of the maxRSSI versus depth in seconds of the measurement set.

Table 7 provides the results (% cell prediction accuracy) of the five aforementioned maxRSS methods, applied to the 20 datasets introduced in Table 6.

Cell prediction accuracy (% percentage), as the ratio of correct predictions to the total number of predictions per method, was adopted as the most important performance indicator (most reported metric in indoor positioning) to aid comparison between diverse methods. The color-code in Table 7 denotes improvement (green), deterioration (pink), or maintaining (gray) of prediction accuracy compared to the basic maxRSS-1 method. The last column on the right provides the seconds for peak maxRSS-X method value.

Table figures are discussed in detail in Section 4.1, and methods are evaluated in relation to the other tables in Section 3.

### 3.3. RSSI Spatial Management

As already mentioned in Section 2.4, RFID, originally intended to be used as a hybrid precision-enhancing technology, was only installed in a small part of the MNEP, due to the size and complexity of the RFID reader/antenna and cables. RFID measurements were only taken at the Tsisdaraki Mosque (Tz) during the pilot tests, while further experiments on the cooperation of the RFID technology with BLE were carried out at the premises of the UoP in a lab environment, where a space similar in terms of layout to the small/medium rooms of the MNEP was created. As RFID cells are smaller by order of magnitude compared to BLE cells, they form a completely different use case, with minimal overlaps when covering the same showcases, and a very high accuracy, despite a low response (reaction time when wristbands enter the active area of an antenna) observed in both the lab and the Tsisdaraki Mosque. For these reasons and considering the additional cost (RFID is three to four times more expensive than BLE), they were put out of the scope of the current work.

According to the VTT architecture in Section 2.1, an additional layer of spatial/topological filtering is applied to detect/correct possible positioning errors and/or failures. Following the same low complexity/cost-efficiency concept, as in previous steps, and considering the “nomadic-structured” model of visitor movement, we adopted a simple approach building single-hop static graphs of permitted transitions between cells, constructed separately for each museum space (building–floor) covered by the system.

**Figure 12:**
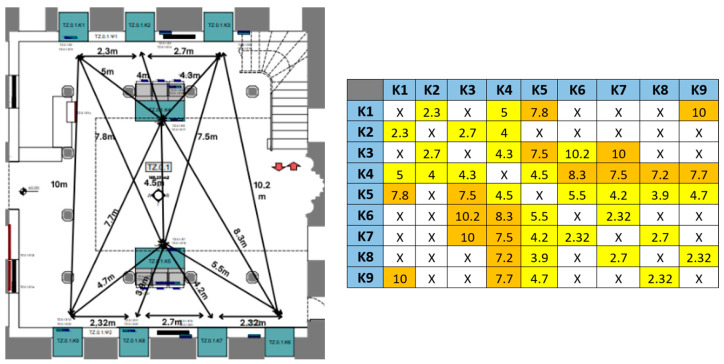
Graph on hall plan and table of permitted transitions (meters) for the Tsisdaraki Mosque.

Figure 12 illustrates the graph and the corresponding table of permitted transitions for the Tsisdaraki Mosque, as an example.

The creation of the graph takes into account the different criteria for defining a transition as permissible or not, including the location and layout of the nodes in the space (i.e., distances between BLE gateways), the shape and size of the room (cell overlaps), and the rationale for exhibit allocation, constituting thematic sets or routes recommended by the Museum. In this sense, the effectiveness of the specific logical composition of the graphs is also evaluated when conducting tests. For example, the original version of the graph considered as permissible the transition from K1 (hall edge) to K5 (hall center) and K9 (opposite hall edge). However, during pilot tests it was observed that the transition from K1 to K5 or K9 is not possible without the visitor being recorded by K4 (central showcase, closer to K1), so the table of permitted transitions was modified accordingly (orange cells). If a cell transition is discarded as non-permissible, the graph method considers the RSSI generating it as a random outlier and retains the last accepted cell prediction. Table 8 provides the results (% cell prediction accuracy) of applying appropriate building/hall graphs on the four maxRSS methods for the 20 datasets in Table 6. The color-code denotes improvement (green), deterioration (pink), or maintaining (gray) of prediction accuracy compared to the corresponding maxRSS method. Graph performance for all methods is evaluated in Section 4.2, in relation to the other tables in Section 3.

### 3.4. ML Classification

According to the VTT functional architecture in Section 2.1, a machine learning (ML) prototype was implemented for current cell prediction, incorporating in its training pilot RSSI measurements taken in each separate cell by applying the three-role measurement pattern described in Section 2.6.

The use of ML classification was carried out with a proof-of-concept, generic approach, without thoroughly exploring all available algorithms, or their potential to optimize the result by fine-tuning their parameters, by cross-scenario testing, or by combining them. The focus was on the overall impact of the environment (hall size and sensor density) and visitor behavior/movement. In this context, representatives of six important ML classification algorithms, widely used according to the recent relevant literature [2,9,11,12,22,23,24,43,44], were selected and tested, using the 20 datasets in Table 6 based on the relevant implementations from the open-source tool Weka (Waikato Environment for Knowledge Analysis is an open-source software suite for machine learning and data mining tasks, developed at the University of Waikato, New Zealand. It is widely used in both an academic and industrial context, providing a collection of visualization tools and algorithms for data analysis and predictive modeling, along with GUIs for easy access to these functions; the MELTOPENLAB ML prototype was implemented using Weka version 3.8.6) [45]. We applied default Weka parameterization with minimal changes, considering prediction accuracy as the principal metric, and assuming a resource-constrained IoT context. Table 9 summarizes the classification models/algorithms used, their main features, and the associated parameterization (critical parameter values and/or variation from the default Weka values).

Regarding the above table, all classifiers, except for K-nn, are considered to be eager learners, building a complete model during training and using it in prediction in place of the actual data; as opposed to lazy learners, storing training data and using it for every new query (prediction) instance. According to the recent relevant literature [22,43], while lazy learners (K-nn) can be effective in dynamic IoT environments, with minimal tuning, quick adaptation to new data and handing of spatial characteristics inherent in RSSI data, their computational demands during the prediction phase may pose challenges, especially as the data volume increases. On the other hand, eager learners, once trained, can offer faster predictions, making them potentially more suitable for real-time IoT applications.

Considering the size (relatively small to moderate number of records) and type (few imbalances due to gateway malfunction, diverse spatial, and time dependent patterns) of our 20 RSSI datasets tested, we adopted powerful and widely used 10-fold cross-validation, [53] maximizing dataset utility, reducing risk of overfitting, accounting for the variability in data patterns distribution across different subsets, and ultimately ensuring that adequate training data is available in each iteration.

Table 10 provides the results (% cell prediction accuracy) of applying the ML algorithms/models in Table 9 to the 20 datasets in Table 6. Algorithm performance and ranking is presented and discussed in detail in Section 4, in relation to the other tables in Section 3.

## 4. Pilot Evaluation

As introduced in [3], the holistic MELTOPENLAB approach integrates different components and technologies, focusing on cost efficiency, availability, compatibility, and scalability, matching the requirements of modern multi-space and high-traffic museums in terms of both visitor experience enhancement and capabilities provided to museum managers/curators. Considering the strategic perspective of cost-efficient integration, solutions adopted at all levels of the MELTOPENLAB architecture, including the visitor tracking tool (VTT), are potentially justified as a trade-off between technical performance and cost/ease of implementation and operation.

Business modeling in the MELTOPENLAB [36], involving cost-benefit and sensitivity analysis, on critical parameters, such as the area of system coverage, the number and type of expected visitors, installation and operation/maintenance cost per technology, the size and penetration of targeted markets, along with the assumption that museums struggle with scarce resources in the post-COVID-19 era [54,55], indicated that sustainable business scenarios are feasible. The basic business scenario features internal rate of return (IRR), exceeding 15% for a 10-year period, on a net present value (NPV) of over 2.3 million Euro, with a break-even point (BEV) of 19 customers (museums) in the MELTOPENLAB system. A critical ingredient for the success of business scenarios was system cost estimated, based on pilot installation and operation in the MNEP premises.

In this context, 1372.29 m^2^ of the MNEP exhibition areas (halls) were covered with 64 BLE sensors (INGICS iGS01S gateways), 15 WiFi APs (Ubiquiti UAP-AC-LITE), and 15 corresponding powerline passthrough adapter pairs (TP-LINK TL-PA4010P), while 100 wearable BLE neckbands (INGICS iBS01) for visitors were used, along with the corresponding networking equipment and consumables, providing a maximum cost of equipment of 8 Euro per m^2^ covered for the BLE solution adopted. For comparison purposes, the corresponding cost of RFID technology coverage in the MNEP (installation restricted to Tzami Tsisdaraki bld. with nine sensors and 100 wearable wristband passive tags) provided a minimum coverage cost of 29.11 Euro per m^2^. Solution affordability and scalability that aids in long-term investment viability should however be justified by performance.

Considering cell-level localization granularity, well-suited for the MNEPs thematic exhibitions, with semantically connected large showcases/clusters of showcases, cell prediction accuracy, a popular metric in indoor positioning, being the ratio of correct predictions to the total number of cell predictions, was adopted as the principal performance indicator to aid the comparison between diverse VTT cell prediction methods, as described in Section 3.

**Figure 13:**
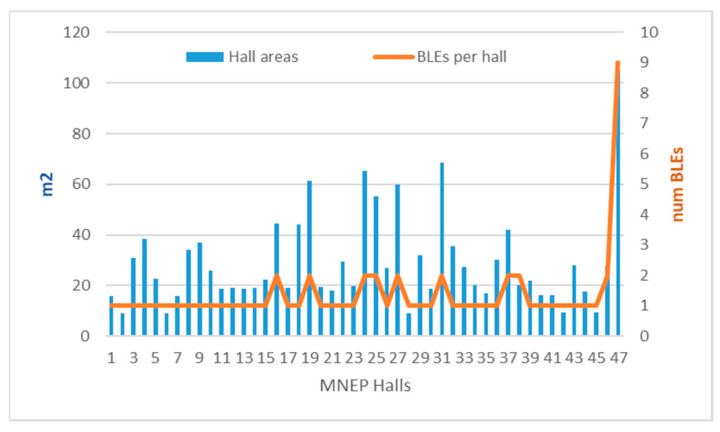
MNEP hall areas distribution vs. BLE gateway (cell) coverage.

An aspect relevant to museum cell planning (in terms of cell size and allocation in space) affecting system cost-efficiency, scalability, and localization granularity is the impact of the density of sensors on system performance. According to Table 1, the area diversity of 47 exhibition halls in eight buildings provides an average hall area of approximately 29.2 m^2^ with a standard deviation of 18.68, corresponding to a sparse sensor placement, an average of one BLE gateway per 23 m^2^ for all MNEP halls, except for one hall (Tz) with relatively dense sensor placement (one BLE gateway/11.69 m^2^), as already mentioned in Section 2.4 and listed in detail in Table 6 (column 4). Considering the adopted cell thresholds (Table 3), providing a theoretical BLE cell area of about 12 m^2^ (wall-mounted gateways) or slightly bigger (ceiling-mounted gateways), the Tsisdaraki Mosque (Tz) was the only case with minimum coverage gaps and maximum cell overlap among all the MNEP halls. Hall area distribution (blue bars) and corresponding cells (orange line) for the MNEP pilot are depicted in Figure 13, illustrating the difference between sparse and dense sensor coverage. Increasing sensor density above the theoretical cell boundaries increases complexity due to RSSI variability, leading to lower accuracy, depending on RSSI management, as discussed in the sequel. In any case, system performance depends on the layout of the halls.

Prior to evaluating the impact of the hall layout and sensor coverage on the VTT performance, the way visitor movement affects datasets should be clarified. As already described in Section 2.5, considering the temporal resolution of RSSI measurements (1 s BLE gateway fastest sampling rate) and the assumed cell roaming rate of visitors (min. 4.6 s), as well the fact that BLE-based systems accumulate delays for processing and denoising of measurements (see Section 3.1), we found that optimal configuration of the VTT tracking (cell refresh) rate, would lie above 2 s and close to 6 s. This range is reflected in the span of RSSI temporal management tests (maxRSS-1, 3, and 6 methods in Section 3.2), and the use of the same *nomadic-structured* measurement pattern (see Section 2.6), in all 20 datasets of Table 6, ensuring coherence with regards to visitor behavior.

In addition to coherent “ant” visitor movement (roaming) between cells, two variations of visitor behavior within a cell were tested, relating to “visitor” and “crowd” roles (see intro of Section 3). As a result, the “.vs” or “.cr” suffix to the name of each dataset constitutes the only dataset variation related to visitor behavior, slow “visitor” vs. fast “crowd” movement patterns. Both, however, were within the above VTT range of operation.

An important issue relating to the reliability of conclusions drawn from the datasets considered is the volume of records. As already mentioned in the introduction of Section 3, BLE gateway malfunctions and problems with the MNEP connectivity and wiring, affecting the measurement continuity and their numbers in the datasets, led to the limitation of datasets considered.

However, the number of records in the retained 20 datasets, with an average of 504.3 and a standard deviation of 219.2 (see Table 6), was deemed adequate for the application of both temporal/spatial methods and ML classification (see Section 3.4).

A factor obviously affecting record volume, was the “visitor” vs. “crowd” role, with “.vs” datasets being consistently larger, compared to “.cr” ones, by an average percentage of 37.21%. This can be interpreted based on the different speed of movement, as the “crowd” role spends most of its time in the cell, quickly changing directions, thus providing shorter intervals for the detection of its beacon from the surrounding BLE gateways.

**Figure 14:**
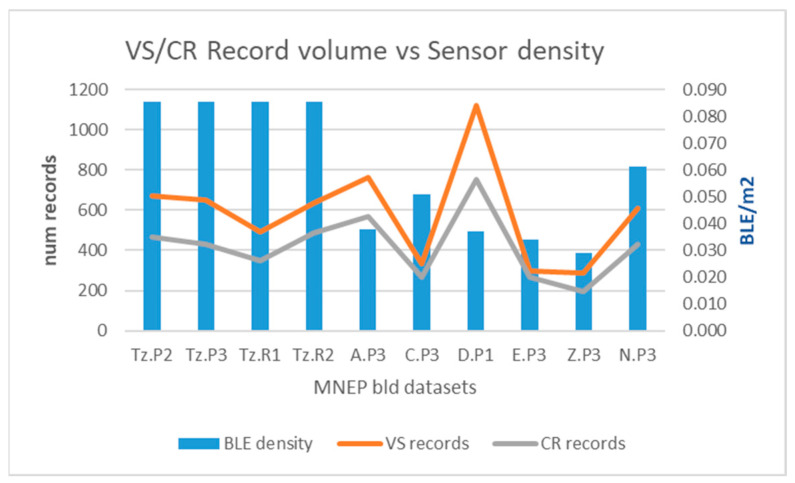
“Visitor”/“Crowd” records volume vs. the MNEP sensor density.

Figure 14 depicts the relationship between the volume of records and the sensor density (BLEs per m^2^—blue bars) for both “visitor” (vs, orange line) and “crowd” (cr, gray line) roles.

According to Figure 14, record volume curves for both roles behave similarly; however, their relationship with sensor density is not coherent. Dense sensor placement (prefix “Tz” datasets) seems to produce a steady number of records, whereas sparse placement produces fluctuations, probably relevant to the characteristics of actual coverage, such as hall layout, distance between cells, etc.

### 4.1. RSSI Temporal Methods Evaluation

**Figure 15:**
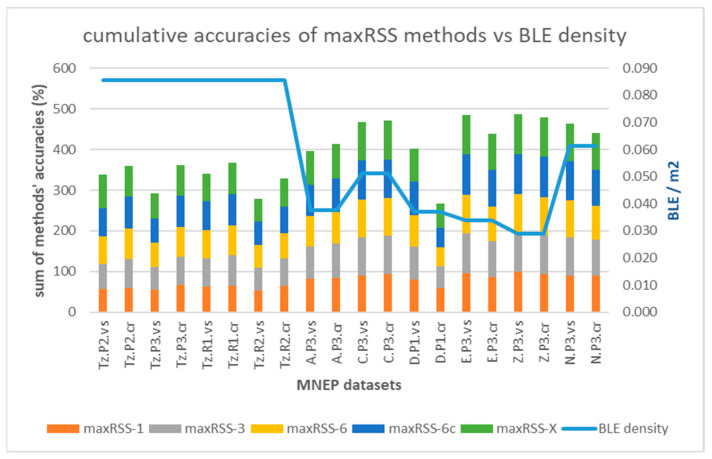
The maxRSS methods cumulative accuracy per dataset vs. BLE sensor density.

Cell prediction based on temporal RSSI methods (Table 7) provided some interesting results. In the first place, accuracies improve when moving from single halls with high sensor density and cell overlap (average 66.64% on all datasets and methods) to tubular-shaped halls with lower density and larger gaps between cells (average 85.25% on all datasets and methods). This trend is depicted in Figure 15, in terms of cumulative accuracy of the five methods (maxRSS-1, 3, 6, 6c, X) per dataset versus the BLE sensor density (line).

Regarding performance comparison between the different methods in Table 7, looking at the color-code, it appears that opening the maxRSS time window from 1 s (min system resolution) to the optimal threshold (6 s), following system logic and visitor modeling, significantly improves accuracy in single rooms with many cell overlaps (green area the upper part of Table 7), while tubular spaces with less overlaps demonstrate smaller improvement, or even deterioration in some cases (lower part of Table 7).

**Figure 16:**
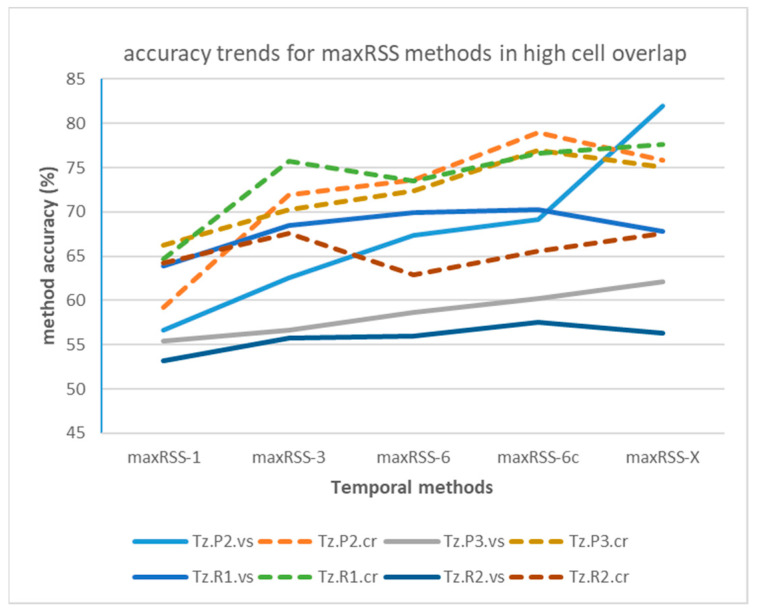
RSSI temporal method accuracy trends in datasets of high cell overlap hall (Tz).

**Figure 17:**
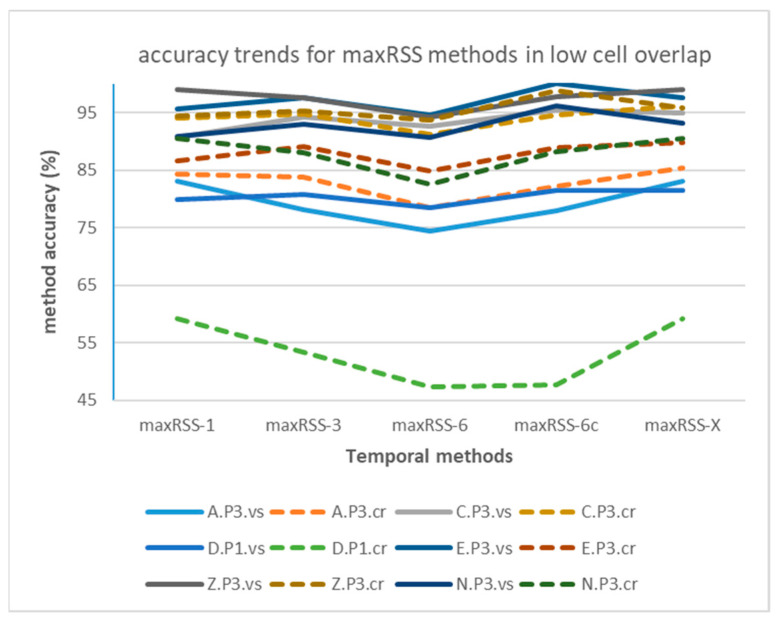
RSSI temporal methods accuracy trends in datasets of low cell overlap halls.

Figure 16 depicts the maxRSS methods accuracy trends in the case of single/high cell overlap hall (Tz), illustrating also the difference (significant improvement) of “crowd” records-based predictions (dotted lines) over “visitor”-based ones (continuous lines). On the other hand, Figure 17 depicts the maxRSS methods accuracy trends in the case of tubular-shaped/low cell overlap halls, illustrating also the difference (little improvement, or mostly deterioration) of “crowd” records-based predictions (dotted lines) over “visitor”-based ones (continuous lines). The lowest performance recorded in the “D.P1.cr” dataset (green dotted curve) is attributed to a different “crowd” role movement at the P1 phase involving a reduced time of staying static in the cell, as compared to other phases.

The method with the best performance in all types of halls/buildings and role-based datasets, according to Table 7, is maxRSS-X, which is no surprise considering that it seeks the best accuracy prediction in a time window spanning over the complete RSSI history in the measurement set. 

**Figure 18:**
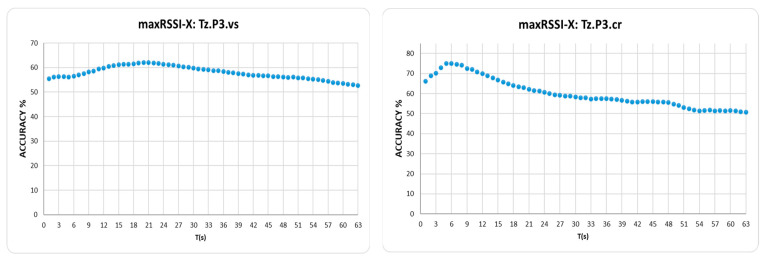
maxRSS-X curves for “visitor”/“crowd” roles in high cell overlap hall (Tz).

However, it is interesting to look into the curves of accuracy maximization (providing the “peak s” in Table 7), depicted indicatively in Figure 18, where “visitor” datasets in high cell overlap hall (Tz) tend to maximize accuracy, or keep it high, for extended RSSI history windows (average 16 s), unlike the “crowd” datasets, maximizing accuracy in 3 s to 5 s, i.e., within the VTT operation range.

**Figure 19:**
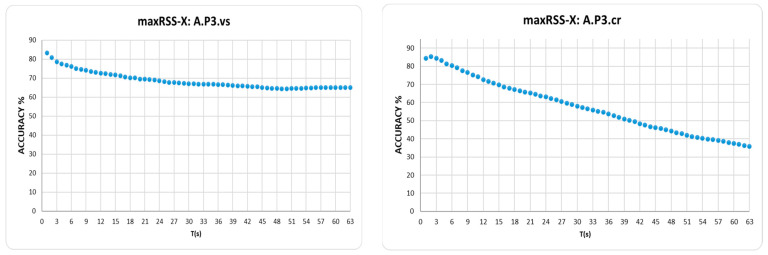
The maxRSS-X curves for “visitor”/“crowd” roles in low cell overlap hall (A).

On the other hand, in tubular-shaped/low cell overlap halls, curves seem to maximize in the first few seconds (average 2 s) for both types of roles, as indicatively depicted in Figure 19.

Considering maxRSS-X as an experiment for the RSSIs time-window span, rather than an actual method that can be practically implemented, the next best performance is provided by maxRSS-6c, a variation of the maxRSS-6 method that discards the first measurements (five records) upon entering a new cell. In this context, border measurements seem to be critical, especially for tubular-shaped/low cell overlap halls, where maxRSS-6c provides a 3.84% average improvement on maxRSS-6, while this improvement is 2.63% on average for the high cell overlap hall. If we also consider practical implementation issues for deciding cell borders, maxRSS-6 is the best method for high cell overlap, while maxRSS-1 performs best for low cell overlap halls.

### 4.2. RSSI Spatial Methods Evaluation

**Figure 20:**
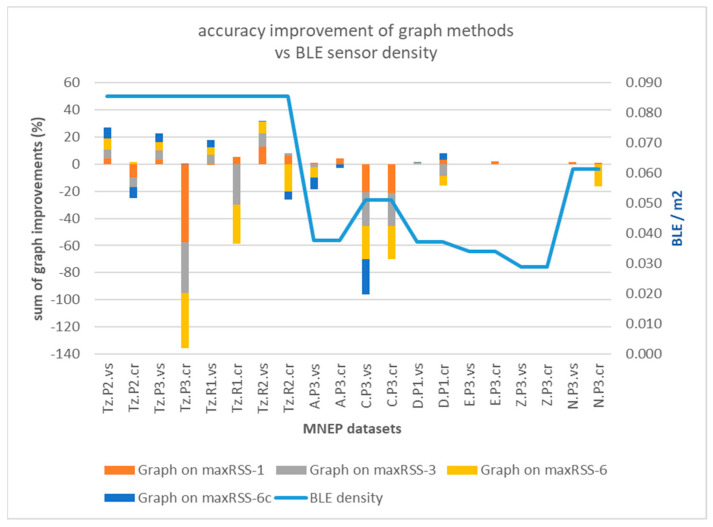
Graph cumulative accuracy improvement vs. BLE sensor density.

Graph-based methods in Section 3.3 provided mixed results, maintaining however the different behavior between the two types of halls, observed in temporal methods (Section 4.1). Figure 20 depicts the graph cumulative accuracy improvement (Table 8) applied on the four methods (maxRSS-1, 3, 6, 6c) per dataset versus the BLE sensor density (line).

**Figure 21:**
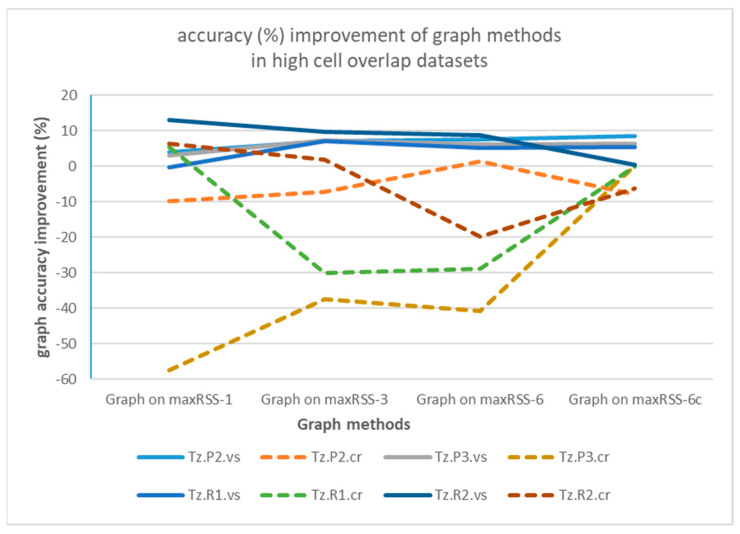
Graph accuracy improvement in datasets of high cell overlap hall (Tz).

**Figure 22:**
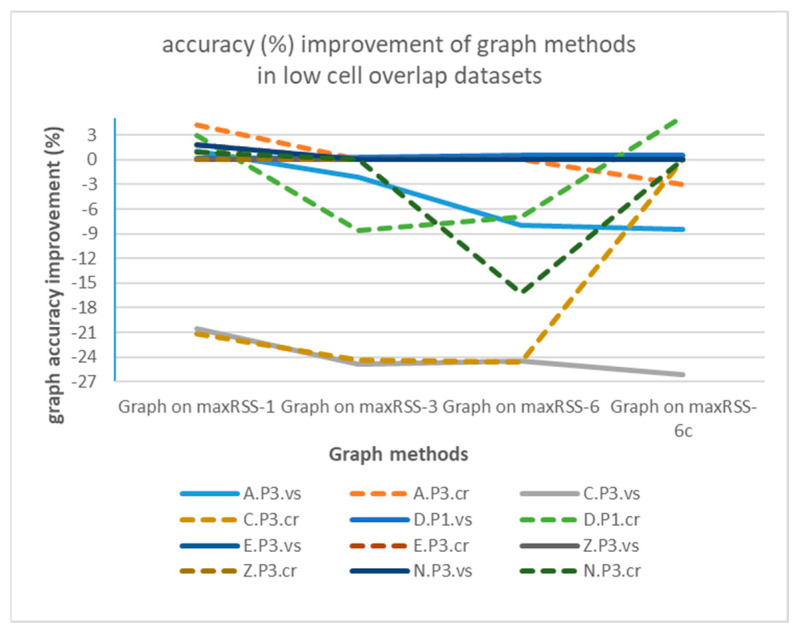
Graph accuracy improvement in datasets of low cell overlap halls.

Graph-based correction on temporal RSS methods seems to be more meaningful for single halls with high sensor density and cell overlap (Tz), providing an increase in accuracy of up to 9.65% (Graph on maxRSS-3 for Tz.R2.vs), nevertheless with dramatic failures for most “crowd”-based datasets. On the other hand, tubular-shaped halls with lower density and larger gaps between cells demonstrate little improvement and deterioration in most cases. The poor performance of “crowd” records-based predictions (dotted lines) over “visitor”-based ones (continuous lines), is better illustrated in Figure 21, for high cell overlap, and in Figure 22, for low cell overlap halls.

Analysis of graph implementation paradigms indicated that, due to its nature (static, single hop), it is sensitive to strong signals (RSSIs) received from sensors that constitute non-permissible transitions. When the correct cell fails to be established in time by a maxRSS method (due to speed/direction of movement, or other spatial factors), application of the graph “locks” prediction to the previous cell, thus propagating error to a number of consecutive cell predictions. This is especially true for tubular halls with low cell overlap, where short signal history (maxRSS-1) provides better prediction over longer time-windows. In high cell overlap halls (Tz), improvement is smaller. Moreover, in both cases, we obtain a significant improvement (4% and 8.43%, respectively) between maxRSS-6 and maxRSS-6c methods, suggesting that graph application enhances maxRSS sensitivity to cell boundaries. Apart from measurements that “lock” graph into consecutive errors, there are also cases where the resumption of the graph function in successful projections is initiated by the occurrence of a “random” (i.e., unexpected) measurement. Such “random” measurements (with positive/negative impact) seem to occur more frequently in “crowd” role datasets, due to more dynamic movement, as well as in tubular spaces, due to the “ant” model sequential movement of the visitor. Last, but not least, graph methods performance is sensitive to gateway malfunctions. To conclude, the realism and efficiency of the representation of spatial layout and visitor movement in the transition graph are critical factors for its added value.

### 4.3. ML Classification Evaluation

Analysis on the ML classification algorithms used and their implementation in Weka is already provided in Section 3.4. Performance (accuracies) presented in Table 10 provide a comprehensive overview for the application of popular ML classifiers, with promising results, as analyzed in the following.

Similar to the behavior indicated for the RSSI temporal methods in Section 4.1, accuracies improve when moving from single halls with high sensor density and cell overlap (average 75.08% on all datasets and algorithms), to tubular-shaped halls with lower density and larger gaps between cells (average 92.90% on all datasets and algorithms—the highest among all accuracies calculated). Probably because reduced cell overlap provides clearer separation for classifiers. ML results outweigh maxRSS methods’ accuracies by an average of 12.67% on high cell overlap and 8.97% on low cell overlap halls.

**Figure 23:**
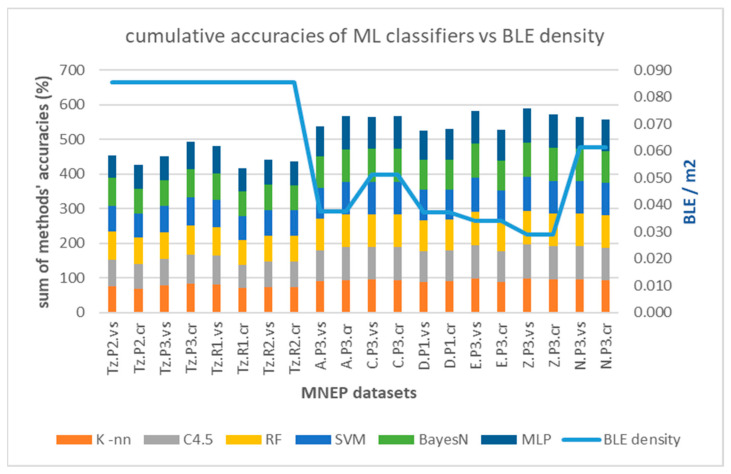
ML classifiers cumulative accuracy vs. BLE sensor density.

This trend is depicted in Figure 23, in terms of cumulative accuracy of the six algorithms (K-nn, C4.5, RF, SVM, Bayes-N, MLP) per dataset versus BLE sensor density (line).

**Figure 24:**
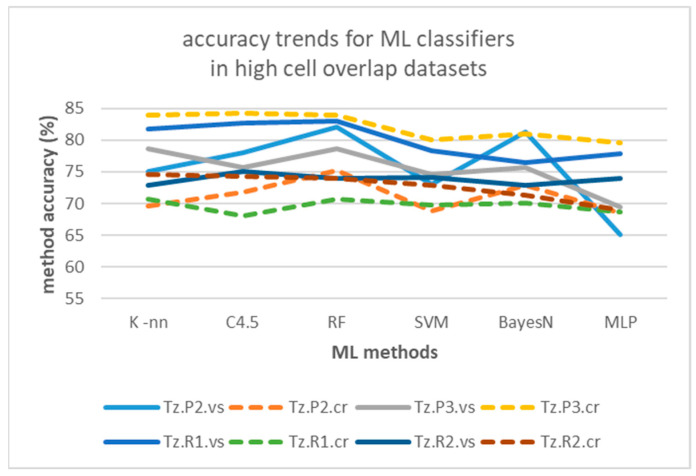
ML classifiers accuracy trends in datasets of high cell overlap hall (Tz).

**Figure 25:**
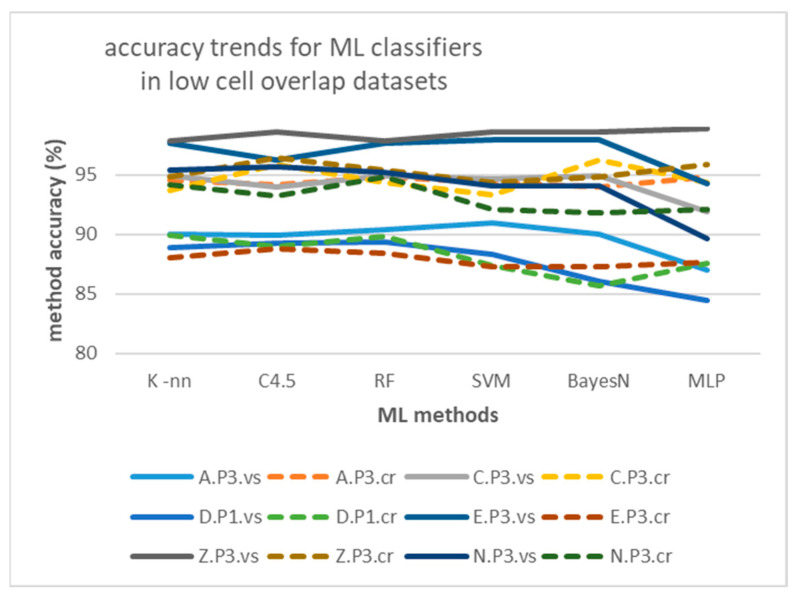
ML classifiers accuracy trends in datasets of low cell overlap halls.

Regarding performance comparison between different algorithms in Table 10, as well as the impact of measurement pattern roles, Figure 24 and Figure 25 depict the relevant trends, respectively, for the high cell overlap hall (Tz) and low cell overlap halls (buildings A, C, D, E, Z, N). It seems that “crowd”-based datasets (dotted lines) perform on average worse than “visitor”-based ones (continuous lines), in contrast to maxRSS methods (Figure 16 and Figure 17). This is probably due to more distinguishable patterns and/or better feature separation in “visitor” records. For the high cell overlap hall, there is a mean difference of 2.34%, which becomes 1.24% for the low cell overlap hall.

However, there are exceptions, such as with dataset Tz.P3.cr, which had the best performance over the high cell overlap hall (Tz) datasets and with all classifiers (max. with K-nn, C4.5 and RF). This dataset was an exception for graph method trends too, providing the overall worst performance, attributed to more dynamic movement of the “crowd” role, that increases “random” measurements, as discussed in Section 4.2.

**Figure 26:**
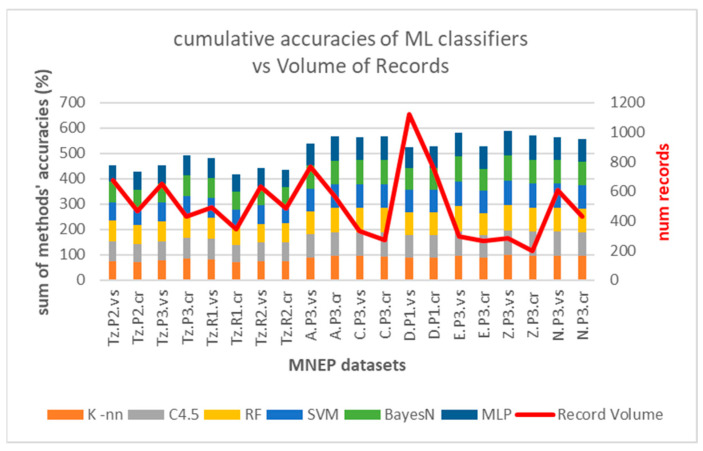
ML classifiers cumulative accuracy per dataset vs. volume of dataset records.

Larger datasets tend to have higher accuracies due to the better statistical representation of data. However, this is only partly true in our case, as datasets with low record volumes still achieve high performance, probably due to low sensor density and clear separations. Figure 26 depicts the performance of ML classifiers versus measurement record volumes.

### 4.4. Ranking of Methods and Algorithms

**Figure 27:**
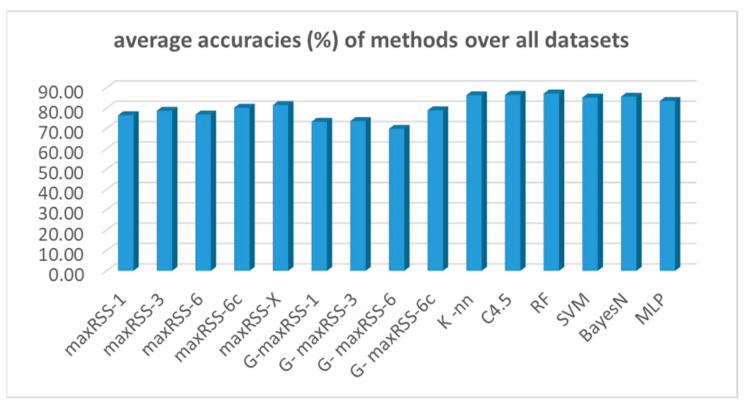
Performance averages of all methods and algorithms.

To provide an overall comparative evaluation, Figure 27 depicts the averages of all prediction methods (nine) and classification algorithms (six) tested.

According to Figure 27, the random forest (RF) classifier had the best average overall performance (87.24%), followed by the C4.5 (86.57%), and the K-nn (86.36%) algorithms. Bayes-N (85.66%), SVM (85.26%), and MLP (83.55%) follow, making ML classifiers the overall winners on top of maxRSS methods (the X version with 81.53%, followed by the 6c version with 80.24% being the best) and graph corrections (G-maxRSS-6c with 78.99% had the best ranking among graph methods).

**Figure 28:**
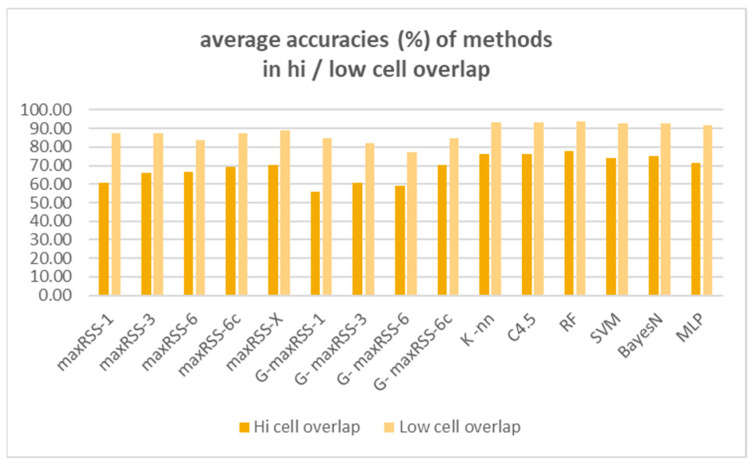
Performance averages of all methods and algorithms in high/low cell overlap halls.

**Figure 29:**
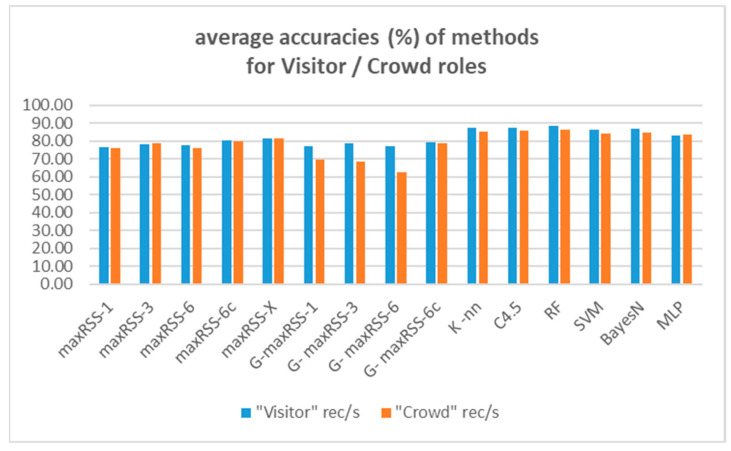
Performance averages of all methods for Visitor/Crowd records.

Having identified cell overlap (relevant to sensor density and hall layout) and measurement pattern role (“visitor”/“crowd”) as important factors affecting the behavior of methods and algorithms, Figure 28 and Figure 29 depict the respective overall averages.

According to Figure 28, method ranking for high cell overlap datasets, is almost the same (with a decrease in rates of 11.63% on average), compared to the overall averages in Figure 26, with minimal rank changes for maxRSS and graph methods. Method ranking for low cell overlap datasets also follows the overall averages ranking in Figure 27, with an increase in accuracy rates of 7.76% on average and minimal rank changes, most interesting of which, is the upgrading (ninth rank) of maxRSS-1 (87.33%). It should also be noted that the low cell overlap datasets average on all methods (88.05%) is almost 20% higher compared to the high cell overlap datasets average (68.66%).

According to Figure 29, method ranking for “Visitor” datasets is almost the same (with an increase in rates of 1.54% on average), compared to the overall averages in Figure 27, with minimal rank changes for maxRSS and graph methods. Method ranking for “Crowd” datasets also follows the overall averages ranking in Figure 27, with a decrease in accuracy rates of 1.54% on average compared to “Visitor” and minimal rank changes, most interesting of which is the upgrading (ninth rank) of maxRSS-3 (78.98%).

**Figure 30:**
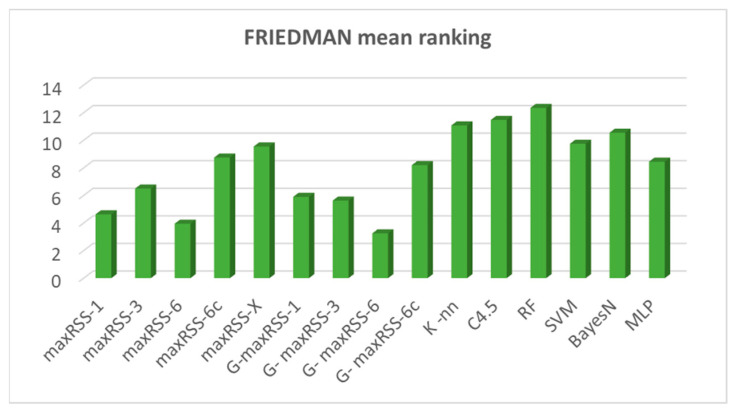
Friedman Test mean ranking of all methods and algorithms.

To conclude with ranking, we conducted the Friedman Test [56,57], a non-parametric statistical method used to detect differences in the performance of multiple algorithms across (three or more) groups of data when data are correlated or repeated measures (e.g., performance scores of classifiers across datasets). It is as an alternative to variance analysis for repeated measures (ANOVA), especially when the assumptions of normality and homoscedasticity are violated. Friedman test is used to detect the existence of statistically significant differences between the mean ranks of classifiers across multiple datasets. It works well with non-normally distributed data, reducing the impact of outliers in the data. It provides a test statistic (p), a low value (<0.05) of which indicates a statistically important difference. In our case, the value was 0.001, thus indicating statistically significant differences. The results of the test, in terms of mean ranking of our 15 methods/algorithms on the 20 datasets, are depicted in the Figure 30.

**Figure 31:**
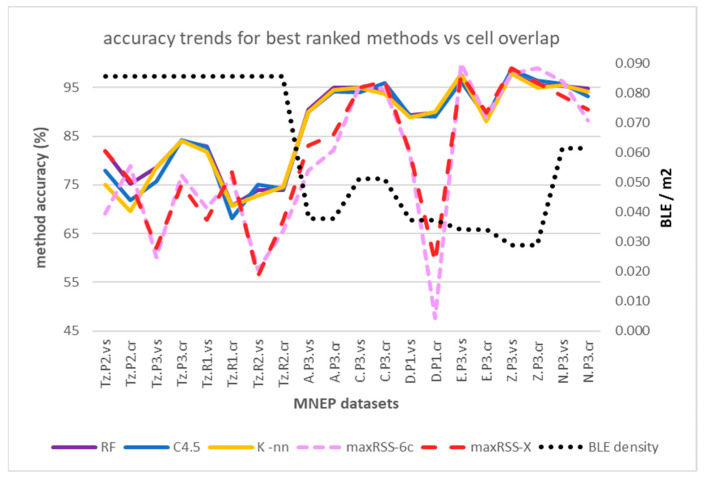
Top ranked algorithms and methods performance (accuracy) across all datasets versus cell overlap (black dotted line).

The best mean rank (12.35) was achieved by the RF classifier, following its performance in all previous comparison tests. As already mentioned in Table 9, being an ensemble learning method, RF combines the merits of both random forests and decision trees, avoiding overfitting, and performing consistently well across diverse dataset patterns. The C4.5 algorithm, with the second-best mean rank (11.48), confirmed the suitability of decision trees-based methods for efficient learning from our datasets. The K-nn algorithm, with a different (much simpler) and effortless philosophy, was ranked third (11.08), providing an efficient adaptive alternative to decision trees. It is worth noting that this ranking (RF, C4.5, K-nn) is maintained in all previous classifications of algorithms, while the performance of the three algorithms across the 20 datasets is quite similar, as illustrated in Figure 31. 

The Bayes-N and SVM algorithms, with mean ranks 10.55 and 9.75, respectively, follow, providing consistent overall performance (fourth and fifth position) in all previous rankings. Multilayer perceptron (MLP) with mean rank of 8.45 (the lowest ML algorithm performance) varies significantly, demonstrating high accuracy in some datasets (e.g., A.P3.cr) but lower in most others. It is at this point that the maxRSS methods come into the picture, with maxRSS-X (mean rank 9.55) and following maxRSS-6c (mean rank 8.75) outweighing MLP. Again, as with the three top classifiers, the performance of the two methods across the 20 datasets is quite similar, as also illustrated in Figure 31, depicting top methods and algorithms performance versus cell overlap.

## 5. Conclusions and Future Work

In the context of the MELTOPENLAB project holistic approach for large cultural institutions, integrating in an innovative way complementary ontologies for digital documentation of exhibits and tools/applications, enhancing both visitor experience and museum performance, we proposed a functional architecture for managing RSSI-based visitor positioning, called the visitor tracking tool. The VTT layered architecture integrates raw RSSI data denoising/smoothing, hybrid positioning technology, temporal methods for visitor cell prediction, spatial filtering, and prediction based on ML classification.

To test the applicability of different solutions and performance of the VTT, we conducted real field experiments in the MNEP, the largest contemporary public museum in Greece, dealing with modern Greek cultural heritage. 

Eight buildings with 47 diverse halls in terms of area and layout, summing up to a total area of 1372.29 m^2^, were covered with BLE sensors, forming virtual cells of coverage, reading RSSI signals from visitor beacons. Cell-level accuracy was found both adequate and feasible, considering semantic connection between showcases/exhibits, resulting in the formation of exhibit thematic clusters assigned to cells. Approximately 130 physical and digital exhibits were mapped to 64 cells, corresponding to a mean density of about two showcases per cell. Due to museum restrictions, limiting RFID installation, as well as relevant high costs, hybrid technology investigation was set out of scope. This, however, will be resumed in future experimentation.

Visitor movement modeling based on critical parameters and influencing signal measurements (RSSI) provided three basic scenarios mapped to popular (in the relevant literature) behavioral models, one of which, “ant”, corresponding to relatively slow nomadic-structured cell roaming, was selected for experimentation. It was assumed to be most realistic for the MNEP traffic and suitable for initial experimentation in a multiparameter environment. Future research will be extended to other more dynamic behavioral models, such as “butterfly” and “fish”. In this context, we proposed and implemented a measurement prototype movement pattern involving four visitors and three roles, two of which were recorded (“visitor” and “crowd”), incorporating different features of movement. Different measurement prototypes for more dynamic movement are already under research.

A total of 34 datasets were produced, including both “visitor” and “crowd” role records. However, due to field implementation issues, including BLE gateway malfunctions, last-minute changes in showcases, and problems with the MNEP connectivity, only 20 datasets were considered valid for production of meaningful results.

Cell prediction accuracy, as the ratio of correct predictions to the total number of predictions per method, was adopted (most reported metric in indoor positioning) to aid comparison between diverse methods.

Pilot implementation decisions and methods adopted at all layers of the VTT architecture followed the overall concept of simplicity, availability, and cost-efficiency, including the open source MQTT lightweight protocol for feeding the BLE gateway data to the VTT, real-time 1D linear Kalman filtering for RSSI equalization (achieving a 76% reduction of variance, and a 51% reduction of standard deviation), RSSI data temporal and spatial management with simple low-processing load methods adopted to visitor movement model (maxRSS), and hall layout (one-hop transition graphs) achieving a maximum prediction accuracy of 81.53% (maxRSS-X) across all datasets. 

Temporal methods and transition graphs are further investigated in a lab environment to confirm the initial findings. In our experimentation approach, we decided to assess temporal and spatial methods separately, considering the overall complexity and multi-parameter problem. However, we intend to provide integration, i.e., collaboration between maxRSS and graph methods, which is the ultimate goal of the overall VTT functional framework.

ML classification was carried out based on RSSI data, as well as spatial hints (e.g., spatially closest cells, or movement-related knowledge, such as next cell when following a specific route). However, in this work we report only RSSI-based results, as spatial hints (effectively a fingerprinting approach) are still under study. We used six popular classification algorithms and their opensource implementation (Weka) without thoroughly exploring all available variations or fine-tuning their parameters, as the focus was on the overall impact of the environment (hall size and sensor density) and visitor behavior/movement. Finally, we adopted powerful and widely used 10-fold cross-validation, maximizing dataset utility, reducing risk of overfitting and accounting for the variability in data patterns distribution across different subsets.

Considering our strategic perspective of cost-efficient integration, pilot choices were justified as a trade-off between technical performance and cost/ease of implementation and operation. In terms of equipment cost, we had a maximum of 8 Euro per m^2^ covered, for the BLE solution adopted. In terms of performance, we had mixed results, mainly affected by cell overlap/hall layout and secondarily by the dynamics of visitor movement.

Increasing sensor density above theoretical cell boundaries increases signal processing complexity due to RSSI variability, thus leading to lower accuracy. Low cell overlap datasets average on all methods (88.05%) was almost 20% higher compared to the high cell overlap datasets average (68.66%).

With regards to movement dynamics, “visitor” role-based datasets had a slightly better average (81.83%) compared to “crowd”, with more dynamic role datasets (78.75%). However, dynamic visitor movement in a cell seems to reduce the value of RSSI history for correct prediction and improve performance in high cell overlap for temporal methods. Dataset size with “visitor” ones consistently larger (37.21% on average), compared to “crowd” ones, did not prove critical, as low record volumes still achieved high performance, especially for low sensor density providing clear separations. Dense sensor placement seems to produce a steady number of records, whereas sparse placement produces fluctuations, probably relevant to characteristics of actual coverage.

Graph correction on maxRSS methods proved more meaningful for high cell overlap halls, providing an increase in accuracy of up to 9.65%, nevertheless with dramatic failures for most “crowd”-based datasets, where dynamic movement increases “random” measurements that destroy graph logic. Measurements taken upon entering or leaving cells, seem to be the most confusing for both temporal and spatial methods. Criteria for creating static graphs incorporating different visitor behaviors, and use of dynamic schemas for spatial filtering are under study, for the next version of the VTT.

With regards to methods and algorithms overall comparative evaluation, ML algorithms outweigh temporal (maxRSS) methods’ accuracies by an average of 12.67% on high cell overlap, and by 8.97% on low cell overlap halls. Friedman test for mean ranking conducted on our 15 methods/algorithms and 20 datasets revealed significant statistical differences and proved RF to be the best algorithm, performing consistently well across diverse dataset patterns (87.24% average accuracy). The C4.5 algorithm, with the second-best mean rank, confirmed the suitability of decision trees for efficient learning from our datasets, while K-nn, with a much simpler and effortless philosophy, was ranked third, providing an efficient adaptive alternative to decision trees. In this context, we intend to proceed with integrating into the VTT a lightweight version of K-nn that utilizes spatial hints dynamically provided by the museum (such as thematic routes), or by visitor statistics (such as popular trajectories), and test it with real visitor foot traffic.

**Figure 32:**
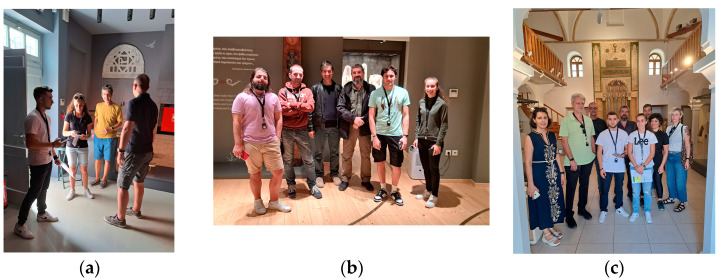
The UoP team (**a**–**c**) and the MNEP personnel (**c**) involved in current work.

Figure 32 depicts the UoP team (a, b, c) and the MNEP personnel (c) involved.

## Figures and Tables

**Table 1:** MNEP room areas distribution (m^2^) per building, coded as A, C, D, E, Z, M, N, and Tz for Tsisdaraki Mosque. Colors range from red (small) to green (large) spaces.

A (m^2^)	C (m^2^)	D (m^2^)	E (m^2^)	Z (m^2^)	M (m^2^)	N (m^2^)	Tz (m^2^)
15.92	18.55	44.56	27	68.4	27.35	22.11	105.23
8.92	19.14	19	60	35.45	20	16.06	
30.75	18.55	44.27	9.2		17	16.19	
38.56	19.14	61.4	32.05		30.28	9.44	
22.62	22.45	19.48	18.79		42	28	
8.91		17.96			20.15	17.6	
16.03		29.6				9.35	
34.02		19.65				27.66	
37.1		65.4					
25.84		55.16					

**Table 2:** BLE beacon and gateway sensor specifications.

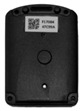	**BLE active beacon (INGICS iBS01)**
	BLE 4.1, 2.4 GHz RF with 2.5 yr battery autonomy (typical)
	PCB omnidirectional antenna with max 30 m range (open space—LOS) and max transmit power +4 dB
	Configurable advertising interval: 100 ms to 10 s
	Dimensions L × W × H (mm): 58 × 42 × 10
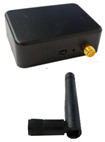	**BLE gateway (INGICS iGS01S)**
	BLE 4.1, 2.4 GHz RF WiFi 802.11 b/g/n(single stream)
	BLE: PCB omnidirectional antenna with max 30 m range WiFi: dipole 2 dBi antenna with max 100 m range
	Bi-direction: Reads message advertised from BLE beacons and/or advertises commands to BLE beacons (by request)
	Support TCP/HTTP(s)/MQTT(s) server
	Dimensions L × W × H (mm): 55 × 41 × 18

**Table 3:** BLE Gateway thresholds per pilot phase and building.

Bld	P1	P2	P3	P4	R1/R2
**A**	-	-	−80 dBm	−80 dBm	-
**C**	-	-	−80 dBm	-	-
**D**	−80 dBm	-	-	−80 dBm	-
**E**	-	-	−80 dBm	−75 dBm	-
**Z**	-	-	−80 dBm	-	-
**M**	-	-	-	−80 dBm	-
**N**	-	-	−80 dBm	-	-
**Tz**	-	−90 dBm (Κ1–Κ5) −75 dBm (Κ6–Κ9)	−75 dBm (Κ1–Κ3) −80 dBm (Κ4–Κ5) −70 dBm (Κ6–Κ9)	−75 dBm (Κ1–Κ3) −80 dBm (Κ4–Κ5) −70 dBm (Κ6–Κ9)	−75 dBm (Κ1–Κ3) −80 dBm (Κ4–Κ5) −70 dBm (Κ6–Κ9)

**Table 4:** Visitor behavioral models based on movement parameters.

	Visitor Behavioral Model		
Movement Parameters	«Ant»	«Butterfly»	«Fish»
**Proximity**			
Visitor in cell			
Visitor in cell boundaries			
Visitor out of cell			
**Contact (sensor—visitor)**			
LOS			
NLOS			
**Cell roaming rate**			
Relatively slow (>24.6 s)			
Relatively fast (<24.6 s)			
**Navigation mode**			
Nomadic–Structured			
Nomadic–Free			
Continuous–Free			

**Table 5:** Evolution of the MELTOPENLAB pilot phases.

P1	P2	P3	P4	R1, R2
1881 records	4800 records	>100,000 records	7446 records	3183 records
D (pattern-2)	Tz (pattern-2)	Tz, A, C, D, E, Z, M, N (pattern-4)	Tz, A, D, E, M (pattern-4)	Tz (pattern-4)

**Table 6:** MELTOPENLAB datasets for *Nomadic_Structured* visitor movement.

#	Dataset ID	MNEP Bld [m^2^, BLEs]	m^2^/BLE	Pilot Phase: BLE Thresholds (dBm)	RECs	RECs/m^2^	Role RECs Used (Pattern)
**1**	Tz.P2.vs	Tzami (105, 9)	11.69	P2: −90, −75	673	6.41	VISITOR (pattern-2)
**2**	Tz.P2.cr	Tzami (105, 9)	11.69	P2: −90, −75	468	4.46	CROWD (pattern-2)
**3**	Tz.P3.vs	Tzami (105, 9)	11.69	P3: −75, −80, −70	650	6.19	VISITOR (pattern-4)
**4**	Tz.P3.cr	Tzami (105, 9)	11.69	P3: −75, −80, −70	432	4.11	CROWD (pattern-4)
**5**	Tz.R1.vs	Tzami (105, 9)	11.69	R1: −75, −80, −70	493	4.70	VISITOR (pattern-4)
**6**	Tz.R1.cr	Tzami (105, 9)	11.69	R1: −75, −80, −70	348	3.31	CROWD (pattern-4)
**7**	Tz.R2.vs	Tzami (105, 9)	11.69	R2: −75, −80, −70	634	6.04	VISITOR (pattern-4)
**8**	Tz.R2.cr	Tzami (105, 9)	11.69	R2: −75, −80, −70	487	4.64	CROWD (pattern-4)
**9**	A.P3.vs	Bld.A (239, 4 + 5)	26.52	P3: −80	765	3.20	VISITOR (pattern-4)
**10**	A.P3.cr	Bld.A (239, 4 + 5)	26.52	P3: −80	568	2.38	CROWD (pattern-4)
**11**	C.P3.vs	Bld.C (98, 2 + 3)	19.57	P3: −80	335	3.42	VISITOR (pattern-4)
**12**	C.P3.cr	Bld.C (98, 2 + 3)	19.57	P3: −80	269	1.13	CROWD (pattern-4)
**13**	D.P1.vs	Bld.D (376, 9 + 5)	26.89	P1: −80	1125	2.99	VISITOR (pattern-2)
**14**	D.P1.cr	Bld.D (376, 9 + 5)	26.89	P1: −80	756	3.16	CROWD (pattern-2)
**15**	Ε.P3.vs	Bld.E (147, 5)	29.41	P3: −80	297	2.02	VISITOR (pattern-4)
**16**	Ε.P3.cr	Bld.E (147, 5)	29.41	P3: −80	268	1.12	CROWD (pattern-4)
**17**	Z.P3.vs	Bld.Z (104, 2 + 1)	34.62	P3: −80	285	2.74	VISITOR (pattern-4)
**18**	Z.P3.cr	Bld.Z (104, 2 + 1)	34.62	P3: −80	196	0.82	CROWD (pattern-4)
**19**	N.P3.vs	Bld.N (146, 4 + 5)	16.27	P3: −80	608	4.16	VISITOR (pattern-4)
**20**	N.P3.cr	Bld.N (146, 4 + 5)	16.27	P3: −80	429	1.79	CROWD (pattern-4)

**Table 7:** MELTOPENLAB *Nomadic_Structured* datasets evaluation (% cell prediction accuracy) of RSSI temporal methods with color-code.

#	Dataset	maxRSS-1	maxRSS-3	maxRSS-6	maxRSS-6c	maxRSS-X	Peak s
**1**	Tz.P2.vs	56.61	62.59	67.37	69.11	82	52 s
**2**	Tz.P2.cr	59.19	71.89	73.65	78.96	75.86	5 s
**3**	Tz.P3.vs	55.38	56.64	58.6	60.17	62.06	21 s
**4**	Tz.P3.cr	66.2	70.23	72.37	77	75	5 s
**5**	Tz.R1.vs	63.89	68.43	69.88	70.2	67.77	10 s
**6**	Tz.R1.cr	64.66	75.72	73.47	76.57	77.57	28 s
**7**	Tz.R2.vs	53.15	55.7	55.96	57.56	56.26	4 s
**8**	Tz.R2.cr	64.27	67.63	62.86	65.61	67.63	3 s
**9**	A.P3.vs	83.14	78.11	74.47	77.92	83.14	1 s
**10**	A.P3.cr	84.33	83.75	78.51	82.2	85.36	2 s
**11**	C.P3.vs	90.75	94.29	92.73	95.48	94.89	3 s
**12**	C.P3.cr	94.05	94.76	91.29	94.67	96.27	2 s
**13**	D.P1.vs	79.82	80.77	78.48	81.52	81.58	2 s
**14**	D.P1.cr	59.13	53.32	47.4	47.67	59.13	1 s
**15**	Ε.P3.vs	95.62	97.63	94.52	100	97.64	2 s
**16**	Ε.P3.cr	86.57	89.1	84.79	88.89	89.81	4 s
**17**	Z.P3.vs	98.95	97.53	94.29	97.78	98.95	1 s
**18**	Z.P3.cr	94.39	95.36	93.72	98.9	95.9	2 s
**19**	N.P3.vs	90.79	93.07	90.71	96.27	93.25	2 s
**20**	N.P3.cr	90.44	88.06	82.55	88.28	90.44	1 s

**Table 8:** MELTOPENLAB *Nomadic_Structured* datasets evaluation (% cell prediction accuracy) of Graph over RSSI temporal methods, with color-code.

#	Dataset	Graph on maxRSS-1	Graph on maxRSS-3	Graph on maxRSS-6	Graph on maxRSS-6c
**1**	Tz.P2.vs	60.62	69.6	75	77.71
**2**	Tz.P2.cr	49.36	64.59	74.95	70.92
**3**	Tz.P3.vs	58.31	63.89	64.65	66.61
**4**	Tz.P3.cr	8.8	32.79	31.62	77.26
**5**	Tz.R1.vs	63.49	75.56	75	75.72
**6**	Tz.R1.cr	70.11	45.66	44.61	76.57
**7**	Tz.R2.vs	66.25	65.35	64.71	57.89
**8**	Tz.R2.cr	70.64	69.48	42.95	59.28
**9**	A.P3.vs	84.18	76.02	66.45	69.45
**10**	A.P3.cr	88.56	83.75	78.51	79.16
**11**	C.P3.vs	70.15	69.37	68.18	69.35
**12**	C.P3.cr	72.86	70.41	66.67	94.67
**13**	D.P1.vs	80	81.03	79.02	82.09
**14**	D.P1.cr	62.04	44.69	40.48	52.92
**15**	Ε.P3.vs	95.62	97.63	94.52	100
**16**	Ε.P3.cr	88.43	89.1	84.79	88.89
**17**	Z.P3.vs	98.95	97.53	94.29	97.78
**18**	Z.P3.cr	94.39	95.36	93.72	98.9
**19**	N.P3.vs	92.6	93.07	90.71	96.27
**20**	N.P3.cr	91.38	88.06	66.27	88.28

**Table 9:** Weka classification models/algorithms and parametrization applied for the MELTOPENLAB *Nomadic_Structured* datasets evaluation (% cell prediction accuracy).

**Algorithm**	**Description**	**WEKA** **Parameters**
**K-nn**	K-nearest neighbors (IBk in Weka) is a non-parametric algorithm of supervised learning, used for classification and regression, and known for its simplicity. It inserts each new input into the appropriate class based on its nearest neighbors. K-nn stores the training data and memorizes the data set, and is thus considered an effortless (lazy) algorithm, performing well in situations where there is no previous knowledge about the data being used [46]. While being simple and effective for small datasets, lazy learners struggle with scalability due to their reliance on the full dataset. Parameter *K* decides the number of neighbors to be considered in the classification. To avoid ties, *K* usually is an odd number and is best calculated using cross-validation. *K* = 1, i.e., use of the nearest neighbor, gave the best results in our tests.	Weka default, *K* = 1
**C4.5**	C4.5 (J48 in Weka) is a decision tree algorithm based on the Iterative Dichotomiser 3 (ID3) algorithm. Decision trees generated can be used for classification and C4.5 is often referred to as a statistical classifier. To obtain the highest classification accuracy, the best feature for separation is the one with the most information for decision making [47]. Parameter *minnumobj* specifies the minimum number of objects that must be present in a node to divide it further. When a value of 1 is used, the model generates leaves with few samples, leading to a more complex tree. Parameter *confidence* determines the level of model confidence about the prediction under consideration. A low value, such as 0.25 used, accepts predictions with a lower certainty, making it more likely to select classes on more nodes.	Weka default, *minnumobj* = 1, *confidence* = 0.25
**RF**	Random forest is an ensemble learning method, used in classification and regression problems, combining the principles of random forests with decision trees, correcting the latter’s habit of overfitting to their training set [48]. It creates a set of trees and lets them vote to find the most popular class when it comes to classification. The law of big numbers prevents RF from overfitting, unless the input is noisy, or the number of trees in the forest is too large. Parameter *number iterations* determines the number of trees that will make up the forest. Each tree is trained on a different subset of the dataset. Increasing the number of trees improves the generalizability of the model at the cost of training time. In our tests, a value of 400 gave the best performance. Parameter *max_depth* determines the maximum depth of each tree. Increasing this may lead to more complex trees better adapted to the training data; however, it is prone to overfitting. Conversely, a small value may lead to simpler trees that generalize better to new data. We had the best performance with a value of 0 providing unlimited depth, i.e., the tree expands until every leaf has data of the same class.	Weka default, *num. iter.* = 400, *max depth* = 0
**SVM**	Support vector machine is a max-margin, supervised learning model, used for classification and regression. It is one of the most studied models, based on statistical learning frameworks, efficiently performing in both linear and non-linear classification [49], being also resilient to noisy data (e.g., misclassified examples). Its goal is to find a superlevel (or multilevel in the case of regression) that separates the training data in the best possible way. In Weka, we used the sequential minimal optimization (SMO) training method [50], optimizing a minimum subset of only two points in each iteration. SMO admits an analytical solution, reducing the time of kernel computing, as it converges very fast, while it performs well with large datasets. Parameter *eps* is used to determine how close to the optimal solution the optimizer stops. A small value means a very accurate solution but may be slower. Default 0.001 was used. Parameter *gamma* determines how far the influence of an individual training example reaches. High values lead to more influence, while with low values (we used default 0), performance depends more on the overall data distribution. Parameter *cost* sets the cost of violating the soft margins during model training. A high value imposes tighter decision boundaries and smaller margins, avoiding training errors, while a lower value allows larger margins and more flexible boundaries. We used one of the default values, i.e., 1.	Weka default, *eps* = 0.001, *gamma* = 0.0, *cost* = 1
**Bayes-N**	Bayes Net (Bayesian networks) is a probabilistic graphical model for classification and inference, based on Bayes’ Theorem of probability. It provides a clear representation of dependencies between variables and can make predictions even when some data points are missing. However, it requires sufficient data to make accurate estimations, while it assumes conditional independence (training class data should be non-correlated), which may not always be true [51]. In Weka, we used the default Bayes algorithm provided (K2-P1-S BAYES) with default *batchSize* (100) and *estimator* (SimpleEstimator—A.0.5)	Weka default *batchSize* = 100
**MLP**	The multilayer perceptron is one of the simplest and most popular types of neural networks for supervised learning tasks, including classification and regression. The basic difference with a single layer perceptron (SLP) is that it solves non-linearly separable problems by learning complex patterns and relationships in data. It provides three layers: input layer (accepting dataset features), hidden layer (neurons processing inputs with appropriate activation functions), and output layer (producing predicted class labels in classification). It may contain more than one hidden layers and approximate any continuous function, given sufficient neurons and layers. However, it is prone to overfitting, especially with small datasets, and requires careful tuning to achieve optimal performance, particularly in resource-constrained environments [52]. Parameter *HiddenLayers* affects the skill of the network to learn and represent complex relationships in the data. It was set to t, use of a single hidden layer with the same number of nodes as the number of input attributes.	Weka default, *HiddenLayers* = t

**Table 10:** MELTOPENLAB *Nomadic_Structured* datasets evaluation (% cell prediction accuracy) by applying the ML algorithms/models presented in Table 9.

#	Dataset	K-nn	C4.5	RF	SVM	Bayes-N	MLP
**1**	Tz.P2.vs	75.03	78	82.02	73.1	81.27	65.08
**2**	Tz.P2.cr	69.65	71.79	75.21	68.8	72.86	68.58
**3**	Tz.P3.vs	78.61	75.69	78.61	74.61	75.69	69.38
**4**	Tz.P3.cr	84.02	84.25	84.02	80.09	81.01	79.62
**5**	Tz.R1.vs	81.74	82.75	82.96	78.29	76.47	77.89
**6**	Tz.R1.cr	70.68	68.1	70.68	69.82	70.11	68.67
**7**	Tz.R2.vs	72.87	75.07	73.97	74.13	72.87	73.97
**8**	Tz.R2.cr	74.53	74.33	73.92	72.89	71.25	68.99
**9**	A.P3.vs	90.06	89.93	90.45	90.98	90.06	87.05
**10**	A.P3.cr	94.54	94.19	94.89	94.36	94.01	94.89
**11**	C.P3.vs	94.92	94.02	94.92	94.62	94.92	91.94
**12**	C.P3.cr	93.68	95.91	94.42	93.3	96.28	94.42
**13**	D.P1.vs	88.88	89.24	89.42	88.35	86.04	84.44
**14**	D.P1.cr	89.94	89.02	89.81	87.43	85.71	87.56
**15**	Ε.P3.vs	97.64	96.29	97.64	97.97	97.97	94.27
**16**	Ε.P3.cr	88.05	88.8	88.43	87.31	87.31	87.68
**17**	Z.P3.vs	97.89	98.59	97.89	98.59	98.59	98.94
**18**	Z.P3.cr	94.89	96.42	95.4	94.38	94.89	95.91
**19**	N.P3.vs	95.39	95.72	95.23	94.07	94.07	89.63
**20**	N.P3.cr	94.17	93.24	94.87	92.07	91.84	92.07

## Data Availability

Datasets used are available upon request.
